# The control of mushroom pathogen *Lecanicillium fungicola* with fungicides and *Bacillus*-based biocontrol treatments during crop trial studies

**DOI:** 10.1186/s12866-025-04356-y

**Published:** 2025-11-20

**Authors:** Joy Clarke, David A. Fitzpatrick, Kevin Kavanagh, Helen Grogan

**Affiliations:** 1 Teagasc, Horticulture Development Department, Ashtown Research Centre, Dublin 15, Ireland; 2 Department of Biology, Maynooth University, Maynooth, Co. Kildare Ireland

**Keywords:** *Agaricus bisporus*, *Bacillus velezensis*, Biocontrol, Dry bubble disease, Metrafenone, Prochloraz, Salting

## Abstract

**Background:**

*Lecanicillium fungicola* is a fungal pathogen of the white button mushroom (*Agaricus bisporus*) and causes dry bubble disease. Due to the recent withdrawal of approval for the most common fungicide prochloraz, only one approved fungicide, metrafenone can be used on mushroom crops within the European Union. Biocontrol uses antagonist bacteria and is being evaluated as a sustainable alternative to fungicides. *Bacillus velezensis* (QST 713) is the active agent in a commercially available biocontrol product, while *B. velezensis* (Kos) is a novel strain. Both have shown antagonistic activity against *L. fungicola in vitro*. The aim of this work was to evaluate the management of dry bubble disease during large scale crop trials using both fungicide and biocontrol treatments and using a range of inoculation levels to establish a level which best reflects on-farm conditions.

**Results:**

An inoculation rate of 1 × 10^4^ conidia m^−2^ applied on day 12 was determined to reflect disease conditions on mushroom farms most closely. At this inoculation rate, the fungicide metrafenone achieved efficacy levels of 96%. Biocontrol treatments Kos and QST 713 were also able to significantly reduce disease development (*p* < 0.05) and resulted in efficacy levels of 74% and 86% respectively. Applying salt to diseased areas on the beds significantly prevented disease outbreak (efficacy 73%), demonstrating that this is a technique which growers should continue to employ.

**Conclusion:**

This work provides important information to the mushroom sector on the treatment of dry bubble disease and provides suggestions to researchers when considering inoculation levels to include for testing biocontrol treatments at a crop level.

**Supplementary Information:**

The online version contains supplementary material available at 10.1186/s12866-025-04356-y.

## Background

Dry bubble disease is a serious concern for growers of the white button mushroom (*Agaricus bisporus* (Lange) [Imbach]). *A. bisporus* is one of the few mushroom species which can be grown commercially and on an industrial scale [1]. Of the 43 million tonnes of cultivated mushrooms produced worldwide between 2018 and 2019, around 11% (4.7 million tonnes) were button mushrooms [2]. Globally *A. bisporus* production ranks fourth behind *Pleurotus ostreatus* (oyster) (16%), *Auricularia auricular* (wood ear) (21%) and *Lentinula edodes* (shiitake) (26%) due to the popularity of these species in the Asian commercial market, but *A. bisporus* is the most popular and commercially grown mushroom species in Europe, Australia and the United States [2, 3]. *A. bisporus* cultivation can be negatively impacted by several diseases which can be caused by either fungal, bacterial, or viral pathogens [4–6]. Disease will have a direct effect on reducing yield for growers and consequently result in significant revenue losses. The four main fungal diseases that affect *A. bisporus* include dry bubble disease (*Lecanicillium fungicola*), wet bubble disease (*Hypomyces perniciosus*), green mould disease (*Trichoderma aggressivum*) and cobweb disease (*Cladobotryum* spp.) [7]. Dry bubble disease is caused by the fungal pathogen *Lecanicillium fungicola* (Preuss) [8] (previously known as *Verticillium fungicola *[9].

**Fig. 1:**
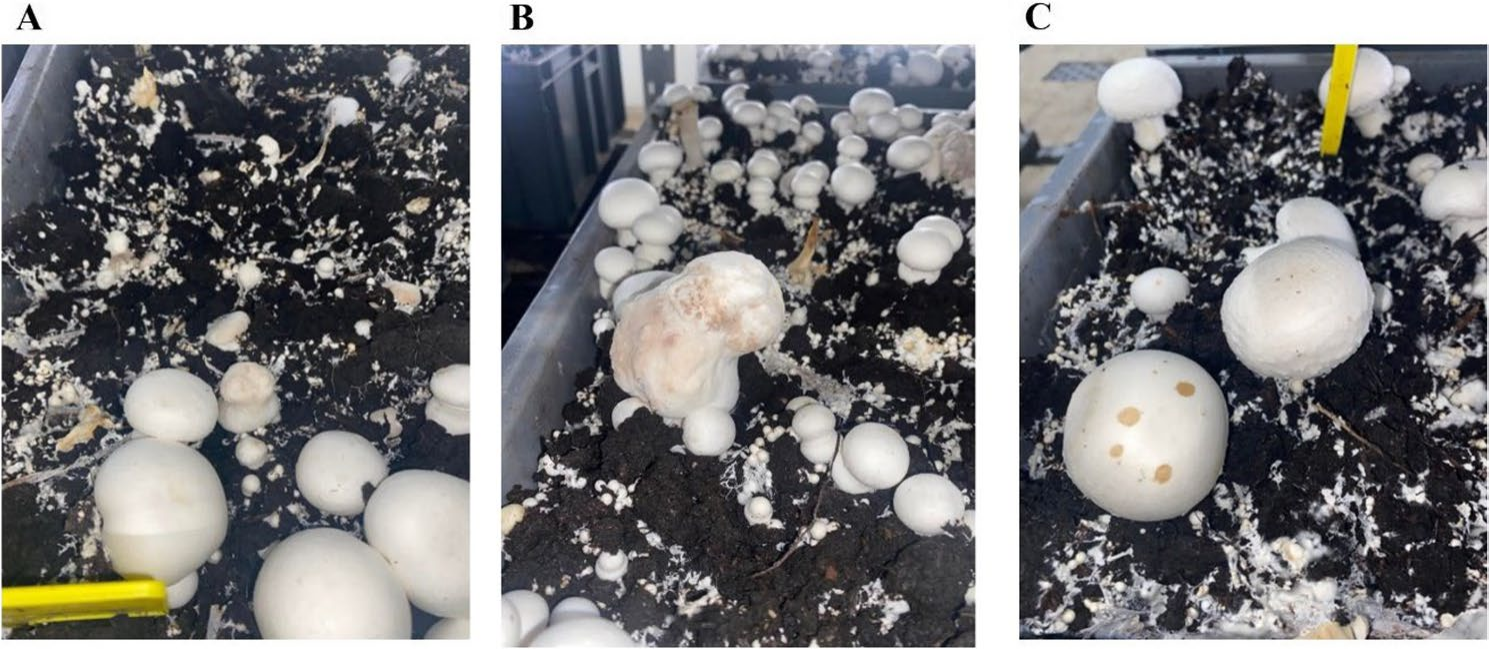
Symptoms of dry bubble disease. **A** early bubble mushroom development, **B** advanced bubble mushroom development and **C** mushroom spotting symptom.

A primary infection occurs when the mushroom pins are infected with the pathogen. The mushroom which emerges will be severely deformed and made up of a large undifferentiated mass of mushroom tissue, this symptom is described as bubble (Fig. 1A-B). Conidia of the pathogen are produced on the infected bubble mushrooms, which are characterised as being easily transferable due to a sticky mucilage covering. The conidia are dispersed by water splash, during crop watering events. Dispersal of the sticky conidia is further aided through their attachment to insect vectors, dust, equipment, pickers’ hands/clothes and many other surfaces [10, 11]. Conidia which land on the cap of developing mushrooms result in the development of spotting symptoms (Fig. 1 C). Another symptom reported for dry bubble disease is stipe blow out, this is generally seen in heavily diseased crops and is characterised by the splitting of stalk tissue [12].

If left untreated, dry bubble disease can result in severely damaged mushroom produce which will directly impact the revenue of growers. One way to control disease levels is to implement strict integrated pest management (IPM) practices on the farm. The eight strategies of IPM include 1: prevention and suppression, 2: monitoring, 3: decision based on monitoring and thresholds, 4: non-chemical methods, 5: pesticide selection, 6: reduced pesticide use, 7: anti-resistance strategies and 8: evaluation [13]. The use of personal protective equipment (gloves, hairnets etc.), foot washes upon entry of growing rooms, and sterilisation of all equipment used is key to limiting disease spread. Mushroom houses must also be well maintained and fitted with door seals. Growers are advised to monitor their crops carefully and identify and treat disease at an early stage before it has the chance to spread. Growers are also encouraged to have a salting routine which involves adding a layer of salt over diseased areas to limit the spread of pathogenic conidia [14]. Most growers also regularly apply preventative synthetic fungicides, which have been a key tool for growers who are dealing with difficulties in controlling diseases. However, fungicide use can have significant effects on non-target organisms and negatively impact human health [15–17]. Growers are also dealing with increased resistance in pathogen populations to the fungicides [5, 18–20]. Prochloraz, a demethylation inhibitor fungicide, was a popular and effective treatment to control diseases, including dry bubble disease, in mushrooms crops [5]. As of June 2023, approval for the use of this fungicide within the European Union (EU) was removed. This left growers in the EU with only one approved fungicide, metrafenone. There is evidence of emerging *Cladobotryum* strains which are tolerant to metrafenone suggesting it will be less effective against cobweb disease [21]. Reports have suggested that *Lecanicillium* isolates are sensitive to the fungicide metrafenone [22, 23]. Currently, there have been no official reports from growers indicating resistance in dry bubble isolates to metrafenone. However further research is needed to assess the sensitivity of *Lecanicillium* to metrafenone.

In recent years there has been a steady decline in the number of approved fungicides, and this has created an urgent need for environmentally sustainable alternatives. This is supported by the European Commission (EC) which outlined a more sustainable approach to pest management in its Sustainable Use of Pesticides Directive (SUD) 2009/128/EC [24]. Biocontrol treatments exploit the antagonistic potential of bacterial strains which are naturally found in the environment [25]. *Bacillus velezensis* species have been investigated as biocontrol strains for several plant crops as they reduce the growth of pathogenic strains through the production of antimicrobial compounds, lytic enzymes or through competition for space and nutrition [26–28]. Biocontrol treatments have also been investigated in relation to mushroom crops [29]. Serenade (AgraQuest Inc.) is a commercially available biocontrol product which contains *B. velezensis* (strain QST 713) as its active agent [30]. This product has been investigated as a potential treatment for several mushroom diseases [30–33]. Another novel biocontrol strain included in this work is *B. velezensis* (strain Kos) which was originally isolated from mushroom casing [34]. This strain has previously been shown to inhibit the pathogens of cobweb disease and dry bubble disease in vitro [35, 36] and has been investigated at a crop level for treatment of cobweb disease [21].

The aim of this work was to investigate the in vitro resistance levels of *L. fungicola* strains towards to prochloraz and metrafenone and to determine the efficacy of both fungicide and biocontrol treatments to control dry bubble disease at a large scale. The optimum experimental inoculation rate which accurately represent disease levels on farms during disease crop trials was also investigated.

## Methods

### Fungal cultures

Two *L. fungicola* strains (620, 1722) were evaluated for their in vitro response to two fungicide active ingredients (a.i): prochloraz and metrafenone. Strain 620 was isolated from an infected mushroom in a commercial crop in 1997, prior to the use of metrafenone. Strain 1722 was isolated from an infected mushroom in a commercial crop in 2020, a few years after metrafenone introduction. Isolate details are shown in Table 1. A small segment of infected mushroom tissue was cultured onto potato dextrose agar (PDA) amended with streptomycin sulfate (100 mg/L) and grown at 25 °C. Both strains were identified based on morphology of the fungus and symptomology on the mushroom crop. The isolates were subsequently preserved in liquid nitrogen and archived in the Teagasc Ashtown culture collection (Dublin, Ireland). Isolate 620 was already known to be sensitive to prochloraz [37] . The pathogenesis of the two isolates has been investigated previously by Quiroz *et al*., (2024) [38].

**Table 1:** *Lecanicillium* isolates used in in vitro experiments

Isolate Number	Species	Year of isolation	Place of origin
620^1^	*L. fungicola*	1997	Surrey, England
1722	*L. fungicola*	2020	Cavan, Ireland

^1^ Grogan *et al*.,(2000) [37]

### Fungicides and biological control agents (BCAs)

The chemical fungicides prochloraz (Sporgon^®^ 50 WP) (460 g a.i kg^−1^) and metrafenone (Vivando^®^) (500 g a.i L^−1^) were obtained from BASF Ireland Ltd. The commercially available biocontrol product Serenade ^®^ ASO (*B. velezensis* QST 713) was obtained from Bayer CropScience Ltd. and contained a minimum of 1 × 10^12^ colony forming units (CFUs) per litre. A bacterial strain *B. velezensis* was originally isolated from mushroom casing [34] (designated here as *B. velezensis* Kos) and was obtained for this work from liquid nitrogen stores at Maynooth University (Kildare, Ireland). Culture filtrate (CF) from this bacterium was produced by inoculating 4 L of sterile nutrient broth (NB) (Thermo Fisher) with 140 h *B. velezensis* Kos liquid culture (1 ml/L). Flasks were grown for 96 h (30 °C at 120 rpm). The CF was collected by centrifugation (1792 x g, 10 min) using a Sorval Lynx 4000 Centrifuge (Thermo Scientific) and F12- 6 × 50 Rotor. The CF was filtered using Miracloth (Merck) into sterile flasks (Duran). These methods have been previously described by Clarke *et al*.,(2024) [21].

### Analysis of in vitro response of Lecanicillium isolates to fungicides

The sensitivity of two *L. fungicola* isolates within the Teagasc Ashtown Culture Collection to fungicides prochloraz and metrafenone was assessed: *L. fungicola* isolate 620 (pre metrafenone introduction) and 1722 (post metrafenone introduction). Cultures were recovered from long term storage and grown on PDA at 25 °C for 5 weeks. Plates were washed with 5 ml PBS + 0.1%v/v TWEEN-20 (VWR Chemicals) and a conidial suspension was collected. The concentration of the conidial suspension was determined using a haemocytometer. The conidial suspension was adjusted with dilutions so that each Sabouraud Dextrose Broth (SDB) flask (50 ml) had a final concentration of 1 × 10^5^ ml^−1^. The flasks were then treated with either prochloraz or metrafenone (1, 10, 100 or 500 mg×kg^−1^). Three replicate flasks were prepared per treatment/isolate combination. Untreated, inoculated flasks were included as a control. Flasks were grown at 25 °C (100 rpm) for 72 h. Fungal mycelium was separated from the liquid with Miracloth and the mycelial wet weight of each flask was determined.

Separately, to determine the effect the various concentrations of prochloraz and metrafenone have on conidiation and hyphal development of the 620 and 1722 isolates, flasks were set up according to the methods outlined above, but using 25 ml SDB and a final concentration of 5 × 10^5^ ml^−1^. After 24 h of growth at 25 °C (100 rpm) evidence of conidiation and hyphal development was monitored using an Olympus microscope (40X). Both isolates were brought forward to be tested in crop trials, however this manuscript will only discuss crop trial results from isolate 1722. Crop trial results from isolate 620 will be detailed in a separate manuscript.

### Mushroom cultivation

Crop trials were carried out in environmentally controlled mushroom growing rooms at the Mushroom Research Unit at Teagasc Ashtown Research Centre (Dublin, Ireland). Plastic crates (external l x b x h dimensions of 400 mm x 600 mm x 300 mm) with a 0.2 m^2^ internal crop surface area was filled with 16 kg (equivalent fill rate of 80 kg/m^2^) of commercially sourced Phase III substrate, spawned with rye grains inoculated with *A. bisporus* strain Sylvan A15 (Carbury Compost Ltd., Carbury, Co. Kildare, Ireland). The crates of substrate were covered with a layer of commercial peat-based mushroom casing (50 mm) (Harte Peat Ltd., Clones, Co. Monaghan, Ireland) on day 1 of the crop cycle and then placed onto shelves in the growing room. Crops were managed following standard operating procedures for mushroom crops in the environmentally controlled growing rooms at the Teagasc Mushroom Unit. Air temperature was set at 21 °C, compost temperature to 25 °C and relative humidity (RH) to a range of 96–100%, for 7 days (case run). After 7 days, fresh air was introduced at 50% and the air temperature and compost temperature were dropped gradually over 72 h to 20 °C and 21 °C respectively (cool down pinning). This change in growing conditions triggers the *A. bisporus* reproductive cycle, resulting in mushroom production. These conditions were maintained for a further 5 days then air temperature was reduced to 18 °C for mushroom harvesting cycles (flushes). Six replicate crates were prepared for each treatment combination. Healthy mushrooms were harvested as predominantly closed cups over two/three flushes and recorded as kg plot^−1^. Diseased or spotted mushrooms were recorded separately. These methods have been previously described by Clarke *et al*.,(2024) [21]. The average number of bubble mushrooms which developed on each plot was recorded for each flush. For crop trial 1, a strict salting regime was undertaken. Once a bubble mushroom had been identified it was recorded, and the area was salted carefully before the crop was watered. If bubble mushrooms were too large to be covered by salt, they were very carefully removed before adding salt to the area on the bed where the bubble mushroom originated from. For crop trial 2 and 3, a separate salting treatment was included where salt was applied in the same manner as described for crop trial 1 only for these specific salted treatment plots. No salt was applied to the control or other treatment plots. In these non-salted plots, bubble mushrooms were recorded and removed carefully only at the end of the flush.

### Crop trials

Three crop trials were conducted to evaluate the efficacy of different fungicides and biological control agents (BCAs) to control dry bubble disease. Crop trial 1 looked at the efficacy of fungicides and BCAs to control dry bubble disease at different rates of inoculation with *L. fungicola* 1722. Crop trial 2 looked at the efficacy of fungicides, BCAs and salting to control dry bubble disease at different rates of inoculation. Crop trial 3 was a repeat of the key treatments in Crop trials 1 and 2 that gave the most interesting results. Crop trials were set up in industry standard growing rooms at Teagasc, Ashtown centre. There were 16, 12 and 12 treatments included, in crop trial 1, 2 and 3 respectively, summarised in Table 2.

**Table 2:** Details of treatments and inoculation rates used in crop trials 1, 2 and 3

Crop trial 1: Efficacy of fungicides and BCAs to control dry bubble disease at different rates of inoculation				
Treatment	Fungicide/BCA/Treatment	Inoculation rate Treatment	L. fungicola strain	Reps
1: Control uninoculated	None	uninoculated	-	6
2: Control 1 × 10^6^ conidia m^−2^	None	1 × 10^6^ conidia m^−2^	1722	6
3: Control 1 × 10^4^ conidia m^−2^	None	1 × 10^4^ conidia m^−2^	1722	6
4: Control 1 × 10^2^ conidia m^−2^	None	1 × 10^2^ conidia m^−2^	1722	6
5: Prochloraz uninoculated	Prochloraz	uninoculated	-	6
6: Prochloraz 1 × 10^6^ conidia m^−2^	Prochloraz	1 × 10^6^ conidia m^−2^	1722	6
7: Prochloraz 1 × 10^4^ conidia m^−2^	Prochloraz	1 × 10^4^ conidia m^−2^	1722	6
8: Prochloraz 1 × 10^2^ conidia m^−2^	Prochloraz	1 × 10^2^ conidia m^−2^	1722	6
9: QST 713 uninoculated	QST 713 (*B. velezensis*)	uninoculated	-	6
10: QST 713 1 × 10^6^ conidia m^−2^	QST 713 (*B. velezensis*)	1 × 10^6^ conidia m^−2^	1722	6
11: QST 713 1 × 10^4^ conidia m^−2^	QST 713 (*B. velezensis*)	1 × 10^4^ conidia m^−2^	1722	6
12: QST 713 1 × 10^2^ conidia m^−2^	QST 713 (*B. velezensis*)	1 × 10^2^ conidia m^−2^	1722	6
13: Kos uninoculated	Kos (*B. velezensis*)	uninoculated	-	6
14: Kos 1 × 10^6^ conidia m^−2^	Kos (*B. velezensis*)	1 × 10^6^ conidia m^−2^	1722	6
15: Kos 1 × 10^4^ conidia m^−2^	Kos (*B. velezensis*)	1 × 10^4^ conidia m^−2^	1722	6
16: Kos 1 × 10^2^ conidia m^−2^	Kos (*B. velezensis*)	1 × 10^2^ conidia m^−2^	1722	6

Crop trial 2: Efficacy of fungicides, BCAs and salting to control dry bubble disease at different rates of inoculation				
Treatment	Fungicide/BCA/Treatment	Inoculation rate Treatment	***L. fungicola*** strain	Reps
1: Control uninoculated	None	uninoculated	-	6
2: Control 1 × 10^4^ conidia m^−2^	None	1 × 10^4^ conidia m^−2^	1722	6
3: Control 1 × 10^2^ conidia m^−2^	None	1 × 10^2^ conidia m^−2^	1722	6
4: Salted uninoculated	Salted	uninoculated	-	6
5: Salted 1 × 10^4^ conidia m^−2^	Salted	1 × 10^4^ conidia m^−2^	1722	6
6: Salted 1 × 10^2^ conidia m^−2^	Salted	1 × 10^2^ conidia m^−2^	1722	6
7: Metrafenone uninoculated	Metrafenone	uninoculated	-	6
8: Metrafenone 1 × 10^4^ conidia m^−2^	Metrafenone	1 × 10^4^ conidia m^−2^	1722	6
9: Metrafenone 1 × 10^2^ conidia m^−2^	Metrafenone	1 × 10^2^ conidia m^−2^	1722	6
10: QST 713 uninoculated	QST 713 (*B. velezensis*)	uninoculated	-	6
11: QST 713 1 × 10^4^ conidia m^−2^	QST 713 (*B. velezensis*)	1 × 10^4^ conidia m^−2^	1722	6
12: QST 713 1 × 10^2^ conidia m^−2^	QST 713 (*B. velezensis*)	1 × 10^2^ conidia m^−2^	1722	6
10: Kos uninoculated	Kos (*B. velezensis*)	uninoculated	-	6
11: Kos 1 × 10^4^ conidia m^−2^	Kos (*B. velezensis*)	1 × 10^4^ conidia m^−2^	1722	6
12: Kos 1 × 10^2^ conidia m^−2^	Kos (*B. velezensis*)	1 × 10^2^ conidia m^−2^	1722	6

Crop trial 3: Efficacy of fungicides, BCAs and salting to control dry bubble disease at different rates of inoculation				
Treatment	Fungicide/BCA/Treatment	Inoculation rate Treatment	***L. fungicola*** strain	Reps
1: Control uninoculated	None	uninoculated	-	6
2: Control 1 × 10^4^ conidia m^−2^	None	1 × 10^4^ conidia m^−2^	1722	6
3: Salted uninoculated	Salted	uninoculated	-	6
4: Salted 1 × 10^4^ conidia m^−2^	Salted	1 × 10^4^ conidia m^−2^	1722	6
5: Metrafenone uninoculated	Metrafenone	uninoculated	-	6
6: Metrafenone 1 × 10^4^ conidia m^−2^	Metrafenone	1 × 10^4^ conidia m^−2^	1722	6
7: QST 713 uninoculated	QST 713 (*B. velezensis*)	uninoculated	-	6
8: QST 713 1 × 10^4^ conidia m^−2^	QST 713 (*B. velezensis*)	1 × 10^4^ conidia m^−2^	1722	6
9: Kos uninoculated	Kos (*B. velezensis*)	uninoculated	-	6
10: Kos 1 × 10^4^ conidia m^−2^	Kos (*B. velezensis*)	1 × 10^4^ conidia m^−2^	1722	6
11: Control 1 × 10^6^ conidia m^−2^	None	1 × 10^6^ conidia m^−2^	1722	6
12: Control 1 × 10^2^ conidia m^−2^	None	1 × 10^2^ conidia m^−2^	1722	6

###  Fungicide and BCA application

For treatment application, the commercial fungicide and BCAs were applied to plots on day 6 after casing (day 1) following the approved rates on the label. Prochloraz was applied at a rate of 1 g of product (Sporgon^®^ 50WP) m^−2^, metrafenone was applied at a rate of 1 ml of product (Vivando^®^) m^−2^ and B. *velezensis* QST 713 was applied at a rate of 0.8 ml of product (Serenade^®^ ASO) m^−2^ (= 0.8 × 10^12^ cfu m^−2^). *B. velezensis* Kos 96 h culture filtrate was prepared fresh on the morning of treatment application. All prepared treatment solutions were applied at a rate of 1 L m^−2^. Water (1 L m^−2^) was applied to control plots. There were two further applications of the two BCA treatments: between 1 st and 2nd flush and again between 2nd and third flush. Water was applied to control and fungicide plots. In crop trial 2 the fungicide Vivando (metrafenone) was used in place of the previously used Sporgon (prochloraz) as the fungicide control treatment. This decision was made due to the imminent expiration of Sporgon approval for use on mushroom crops in the EU from 30th June 2023.

### Crop inoculation

For crop trial 1, 2 and 3, inoculum was prepared for *L. fungicola* isolate 1722. Subcultures of the isolate were grown on PDA at 25 °C for 5 weeks. Plate cultures were washed with PBS + 0.1%v/v TWEEN-20 to collect a concentrated conidial suspension, and the concentration was determined using a haemocytometer. Inoculum for the crop trials was prepared by dilution to give a conidia concentration of 1 × 10^6^ ml^−1^. This was further diluted to give inoculum concentrations of 1 × 10^4^ ml^−1^ and 1 × 10^2^ ml^−1^. A 50 ml aliquot of inoculum of isolate 1722 was applied to each 0.2 m^−2^ plot to give a final application rate of either 1 × 10^6^, 1 × 10^4^ or 1 × 10^2^ conidia m^−2^ according to the crop plan (Table 2). Inoculation of plots took place on day 12 of the crop cycle for crop trial 1 and 2, and on day 11 for crop trial 3.

### Disease data collection

During crop trial 1, a disease assessment for symptomatic bubble mushrooms on plots was carried out regularly over the course of each flush. Any bubble mushrooms found on plots were recorded and salt was carefully applied to cover the infected bubble to limit cross contamination between plots. For crop trial 2 and 3, disease treatment was revised based on the results of crop trial 1. Crop trial 2 and 3 included a specific salting treatment. During these trials, a disease assessment for symptom bubble mushrooms on plots was carried out only at the end of each flush allowing bubble to develop during the flush. Any sizeable bubble mushrooms found at the end of the flush were recorded and were removed carefully to limit cross contamination, but no salt was applied. For the salted treatments, bubble mushroom development was monitored and any bubble mushrooms found at the end of each flush were recorded and salt was carefully applied to cover the infected bubble. Disease incidence was represented by the average number of bubble mushrooms per treatment at the end of the crop trial. Treatment efficacy was calculated using Abbotts formula (Abbott 1925) given as % efficacy = [(Ic -It)/Ic] x 100, where Ic = Disease incidence in the inoculated control; It = Disease incidence in the treated samples [33].

### Statistical analysis

In the in vitro fungicide tests and crop trial studies, after determining normality and equal variance, data were analysed using analysis of variance (ANOVA) in Minitab (version 20.04.00). Differences between treatments were determined using Tukey method and 95% confidence for pairwise comparisons (*p* < 0.05). In the crop trials, treatment plots were arranged on shelves in a randomized block design. During crop trial 2, one plot inoculated with *L. fungicola* 1722 1 × 10^4^ conidia m^−2^ resulted in abnormal disease levels which were not in line with the other replicates. Therefore, disease data analysis for the 1 × 10^4^ conidia m^−2^ plots in crop trial 2 were analysed using 5 replicates rather than 6 to remove this outlier.

## Results

### Analysis of in vitro response of Lecanicillium isolates to fungicides

**Fig. 2:**
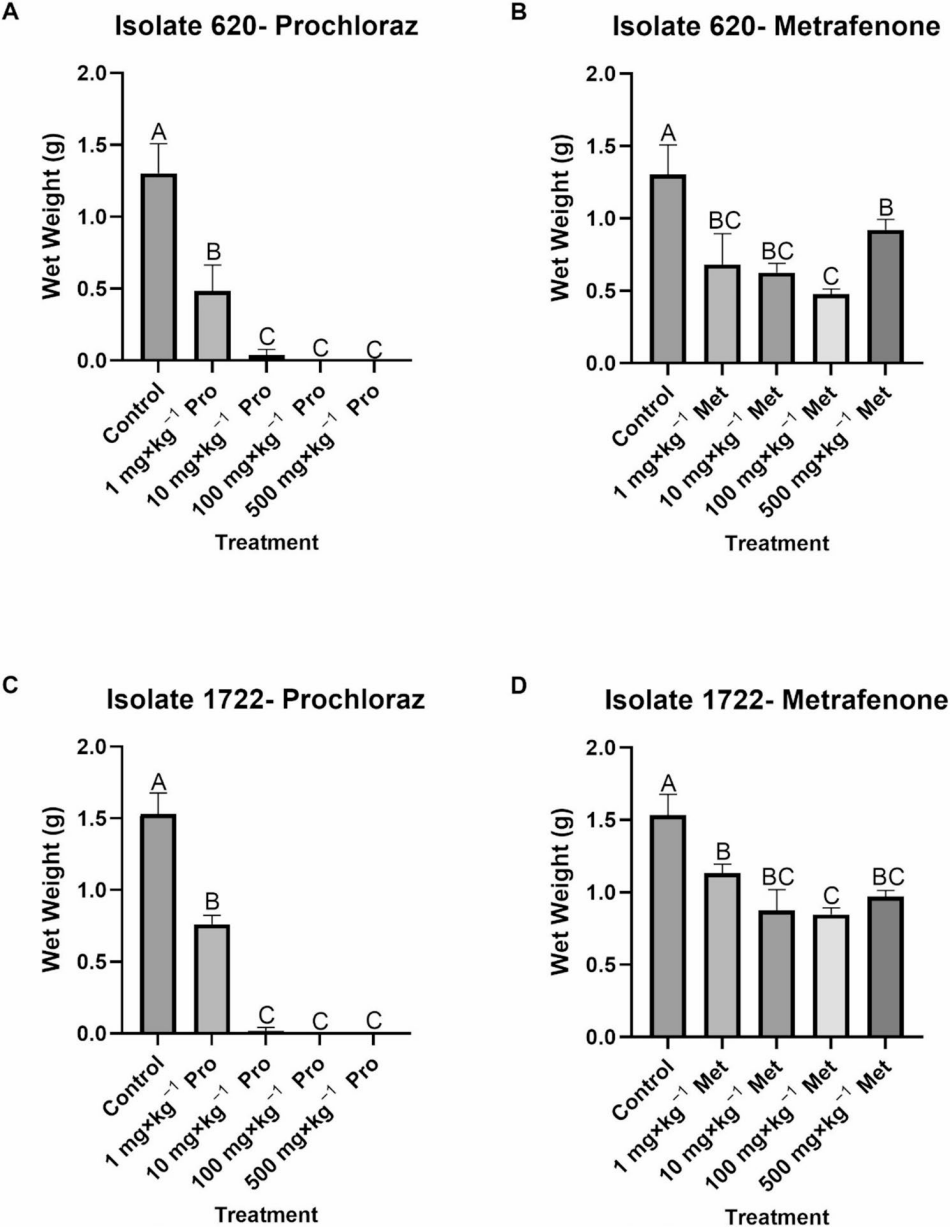
Growth of *Lecanicillium fungicola* in SDB liquid culture **A** isolate 620 with prochloraz (1, 10, 100 or 500 mg×kg^−1^), **B** isolate 620 with metrafenone (1, 10, 100 or 500 mg×kg^−1^), **C** isolate 1722 with prochloraz (1, 10, 100 or 500 mg×kg^−1^) and **D** isolate 1722 with metrafenone (1, 10, 100 or 500 mg×kg^−1^), Data represent the average wet weight of 3 replicates after 72 h for each treatment. Error bars represent standard deviation. Data analysed by ANOVA, *n* = 3. Means sharing the same letter are not significantly different at *P* < 0.05 by Tukeys pairwise comparisons test

Prochloraz was very effective at reducing the growth of isolate 620. At 1 mg×kg^−1^ growth was reduced by 63% while at 10 mg×kg^−1^ growth was reduced by 97%. No growth was recorded for isolate 620 grown in the presence of 100 and 500 mg×kg^−1^ prochloraz **(**Fig. 2A**).** Hyphal development for isolate 620 at 24 h was seen only at 1 mg×kg^−1^ prochloraz (Figure S1 A). Metrafenone also significantly reduced the growth of isolate 620 but growth was less severely affected compared to prochloraz treated flasks. Growth was reduced by 48%, 52%, 63% and 29% for 1, 10, 100 and 500 mg×kg^−1^ respectively (Fig. 2B) and conidiation and hyphal development was observed at all tested concentrations of metrafenone at 24 h (Figure S1 A).

Prochloraz significantly reduced the growth of isolate 1722 in flask cultures, similar to isolate 620. At 1 mg×kg^−1^ growth was reduced by 50% while at 10 mg×kg^−1^ growth was reduced by 99%. No growth was recorded for isolate 1722 grown in the presence of 100 and 500 mg×kg^−1^ prochloraz (Fig. 2C). Hyphal development for isolate 1722 at 24 h was seen only in 1 and 500 mg×kg^−1^ prochloraz (Figure S1 B). Metrafenone reduced the growth of isolate 1722 by 26%, 43%, 45% and 37% for 1, 10, 100 and 500 mg×kg^−1^ metrafenone respectively (Fig. 2D). Conidiation and hyphal development were observed at all concentrations of metrafenone after 24 h (Figure S1 B).

### Crop trial 1: Efficacy of fungicides and BCAs to control dry bubble disease at different rates of inoculation

#### Yield

**Fig. 3:**
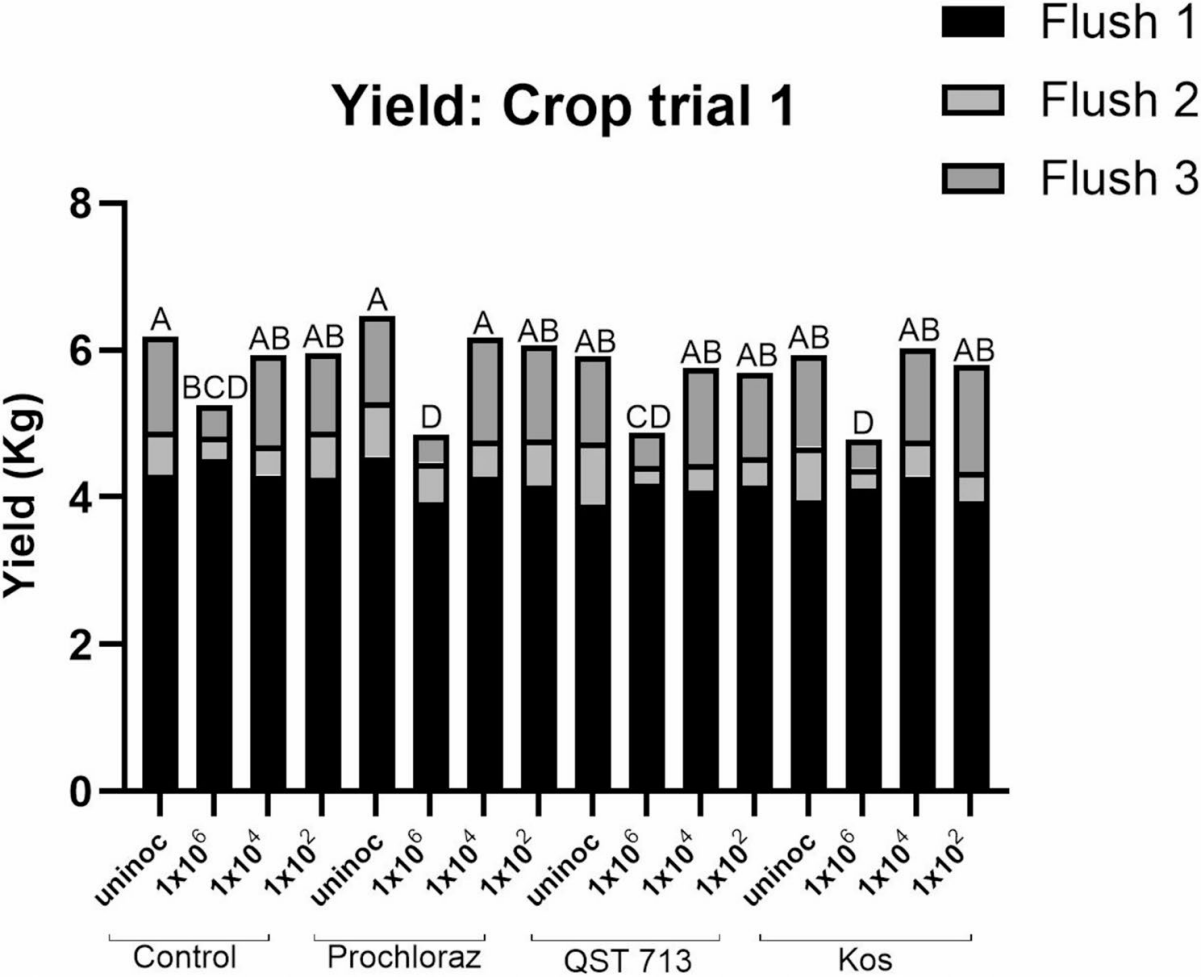
Average healthy yield of *A. bisporus* over three flushes following treatment with the fungicide prochloraz or the BCAs QST 713 or Kos, followed by inoculation with *L. fungicola* 1722 at inoculation rates of either 1 × 10^6^ conidia m^−2^, 1 × 10^4^ conidia m^−2^ or 1 × 10^2^ conidia m^−2^. Data analysed by ANOVA, *n* = 6. Means sharing the same letter are not significantly different at *P* < 0.05 by Tukeys pairwise comparisons test

The average yield of healthy mushrooms for treatments 1–16, collected over three flushes in crop trial 1 can be seen in Fig. 3. There was no statistically significant difference in yield between treatments during flush 1 and flush 2. The yield of flush 1 ranged from 3.9 to 4.5 kg plot^−1^, while during flush 2 the yield was much lower ranging between 0.2 and 0.8 kg plot^−1^. This may be due to a high number of smaller mushrooms being harvested during flush 1 which reflects the high yield recorded during this time and may have negatively impacted the yield for flush 2. The yield for flush 3 ranged from 0.4 to 1.5 kg plot^−1^ and at this point there was a significant difference in yield between treatments. The control inoculated at a rate of 1 × 10^6^ conidia m^−2^ *L. fungicola* and all treatment plots inoculated at this rate were significantly reduced in yield compared to the uninoculated control (*P* < 0.05). For the control inoculated at the two lower inoculation rates (1 × 10^4^ conidia m^−2^ and 1 × 10^2^ conidia m^−2^) and all treatment plots inoculated at these rates, there was no significant reduction in yield compared to the uninoculated control. Total yield over three flushes for the uninoculated controls across all treatments ranged from 5.9 to 6.5 kg plot^−1^. The average yield of each treatment harvested during of trial 1 can be found in Table S1.

#### Dry bubble disease

In crop trial 1 at the end of the first flush, a small number of bubble mushrooms were present (≤ 4 bubbles/plot). These were predominantly on treatments inoculated with 1 × 10^6^ conidia m^−2^ *L. fungicola*. An occasional bubble mushroom was also detected on some 1 × 10^4^ conidia m^−2^ inoculated plots at the end of the first flush, but no bubble mushrooms were found on any 1 × 10^2^ conidia m^−2^ inoculated or any uninoculated plots at this time (Table S2). During flush 2, the number of bubble mushrooms observed on all 1 × 10^6^ conidia m^−2^ inoculated plots had increased considerably but there was still no significant difference between the inoculated control and any of the treatments inoculated at the 1 × 10^6^ conidia m^−2^ rate. The average number of bubble mushrooms developing ranged from 25 to 32 bubbles/plot. A few bubble mushrooms were present in both the 1 × 10^4^ conidia m^−2^ and 1 × 10^2^ conidia m^−2^ inoculated plots, but their numbers were much lower (< 4 bubbles/plot) compared to the 1 × 10^6^ conidia m^−2^ rate (Table S2). Bubble mushrooms were found occasionally on uninoculated plots during flush 2, with < 1 bubble/plot on average. During flush 3, there was minimal bubble mushroom development for the entire crop. There was no significant difference between control treatments and any other treatment group at all three inoculation levels.

**Fig. 4:**
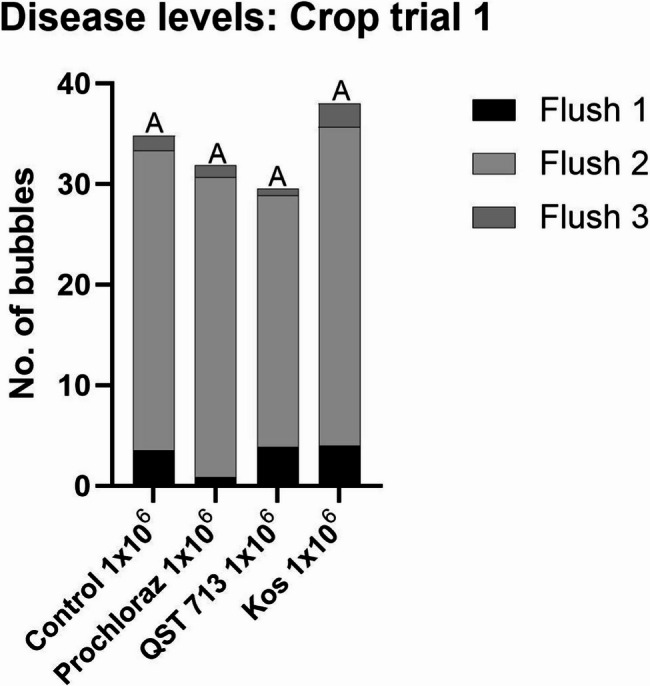
Average number of bubbles recorded at the end of crop trial 1 for plots treated with the fungicide prochloraz or the BCAs QST 713 or Kos, followed by inoculation with 1 × 10^6^ conidia m^−2^ *L. fungicola* 1722. Data analysed by ANOVA, *n* = 6. Means sharing the same letter are not significantly different at *P* < 0.05 by Tukeys pairwise comparisons test

Over the three flushes of crop trial 1 there was significant disease development only on 1 × 10^6^ conidia m^−2^ inoculated plots. The inoculated control plots had a total average of 35 bubbles/plot at the end of the trial while the inoculated plots treated with different products had total averages of between 29 and 38 bubbles/plot. There was no significant difference in disease levels with any of the treatments at the 1 × 10^6^ conidia m^−2^ inoculation rate (Fig. 4). The disease incidence on the 1 × 10^4^ conidia m^−2^ and 1 × 10^2^ conidia m^−2^ inoculated plots remained low in control plots at the end of crop trial 1. There was an average of 3 bubbles on control plots treated with 1 × 10^4^ and no significant difference between control and treatment plots inoculated at the same rate. Control plots inoculated with 1 × 10^2^ conidia m^−2^ had an average of 5 bubbles/plot while inoculated treatment plots had averages of 2 bubbles/plot or less (Figure S2). Disease development for crop trial 1 is summarised in Table S2.

### Crop trial 2: Efficacy of fungicides, BCAs and salting to control dry bubble disease at different rates of inoculation.

#### Yield

**Fig. 5:**
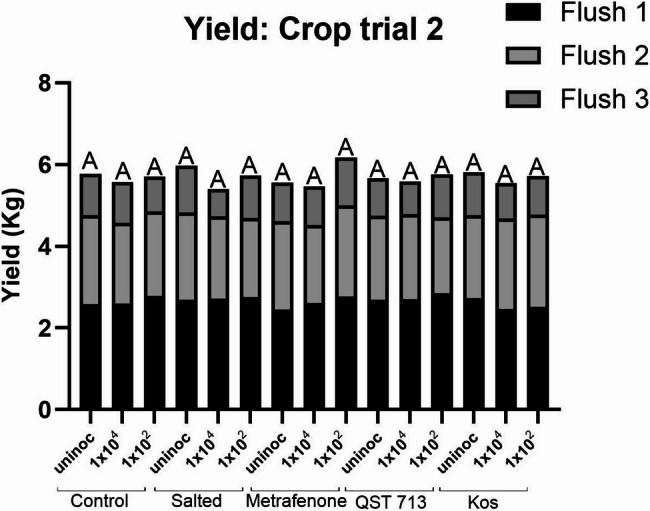
Average healthy yield of *A. bisporus* over three flushes following treatment with salt, the fungicide metrafenone or the BCAs QST 713 or Kos, followed by inoculation with *L. fungicola* 1722 at inoculation rates of either 1 × 10^4^ conidia m^−2^ or 1 × 10^2^ conidia m^−2^. Data analysed by ANOVA, *n* = 6. Means sharing the same letter are not significantly different at *P* < 0.05 by Tukeys pairwise comparisons test

The average yield of healthy mushrooms collected over three flushes following inoculation at rates of 1 × 10^4^ conidia m^−2^ and 1 × 10^2^ conidia m^−2^ *L. fungicola* 1722 during crop trial 2 can be seen in Figure 5. The average yield ranged from 2.4 to 2.85 kg plot^−1^ for flush 1, 1.85 to 2.26 kg plot^−1^ for flush 2 and 0.66 to 1.18 kg plot^−1^ for flush 3. Over the course of this crop trial, there was no statistically significant difference in the yield harvested from the uninoculated control plots with any other treatment/inoculation combination used. Total yield over three flushes for the uninoculated controls across all treatments ranged from 5.56 to 5.97 kg plot^−1^. The average yield of each treatment harvested at the end of trial 2 can be found in Table S3.

#### Dry bubble disease

**Fig. 6:**
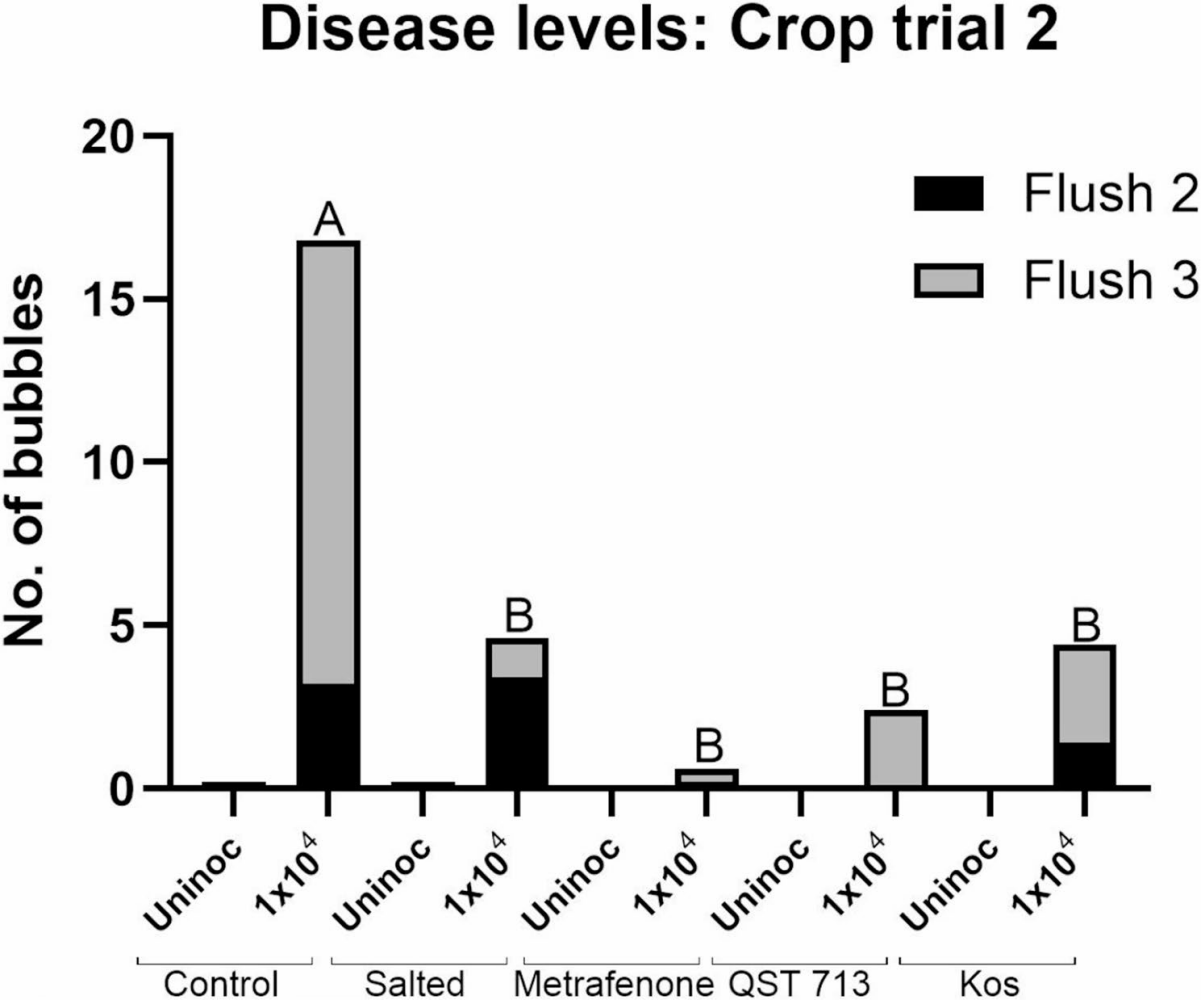
Average number of bubbles recorded at the end of crop trial 2 for plots treated with salt, the fungicide metrafenone or BCAs QST 713, Kos, followed by inoculation with 1 × 10^4^ conidia m^−2^ *L. fungicola* 1722. Data analysed by ANOVA, *n* = 5. Means sharing the same letter are not significantly different at *P* < 0.05 by Tukeys pairwise comparisons test

No bubble mushrooms were recorded during the first flush of crop trial 2. For plots inoculated with 1 × 10^2^ conidia m^−2^ *L. fungicola*, very few bubble mushrooms developed and these were predominantly on the inoculated control plots in the third flush (average 1.5/plot). No bubble mushrooms were recorded for any salted, metrafenone, *B. velezensis* QST 713 or Kos treated plots inoculated at the same rate. Bubble mushrooms appeared on plots inoculated with 1 × 10^4^ conidia m^−2^ *L. fungicola* during flush 2 (Table S4). The highest average number of bubble mushrooms occurred on control plots inoculated with 1 × 10^4^ conidia m^−2^ *L. fungicola* (17 bubbles/plot) (Fig. 6). The average numbers of bubble mushrooms on all treated plots inoculated at the same rate were significantly lower than the control (*p* < 0.05) at < 5 bubbles/plot. The efficacy of the treatments ranged from 73% for salting, followed by 74% and 86% for *B. velezensis* Kos and QST 713, respectively, and 96% for metrafenone (Table S4).

### Crop trial 3: Efficacy of fungicides, BCAs and salting to control dry bubble disease

Crop trial 3 was a repeat of the key treatments in Crop trials 1 and 2 to confirm the results. The main treatments included were: Control (untreated and uninoculated) and Control inoculated at 1 × 10^2^ conidia m^−2^, 1 × 10^4^ conidia m^−2^ and 1 × 10^6^ conidia m^−2^ *L. fungicola* 1722; and the four treatments: salted, metrafenone, QST 713 and Kos, uninoculated and inoculated at 1 × 10^4^ conidia m^−2^ *L. fungicola* 1722 (Table 2).

#### Yield

**Fig. 7:**
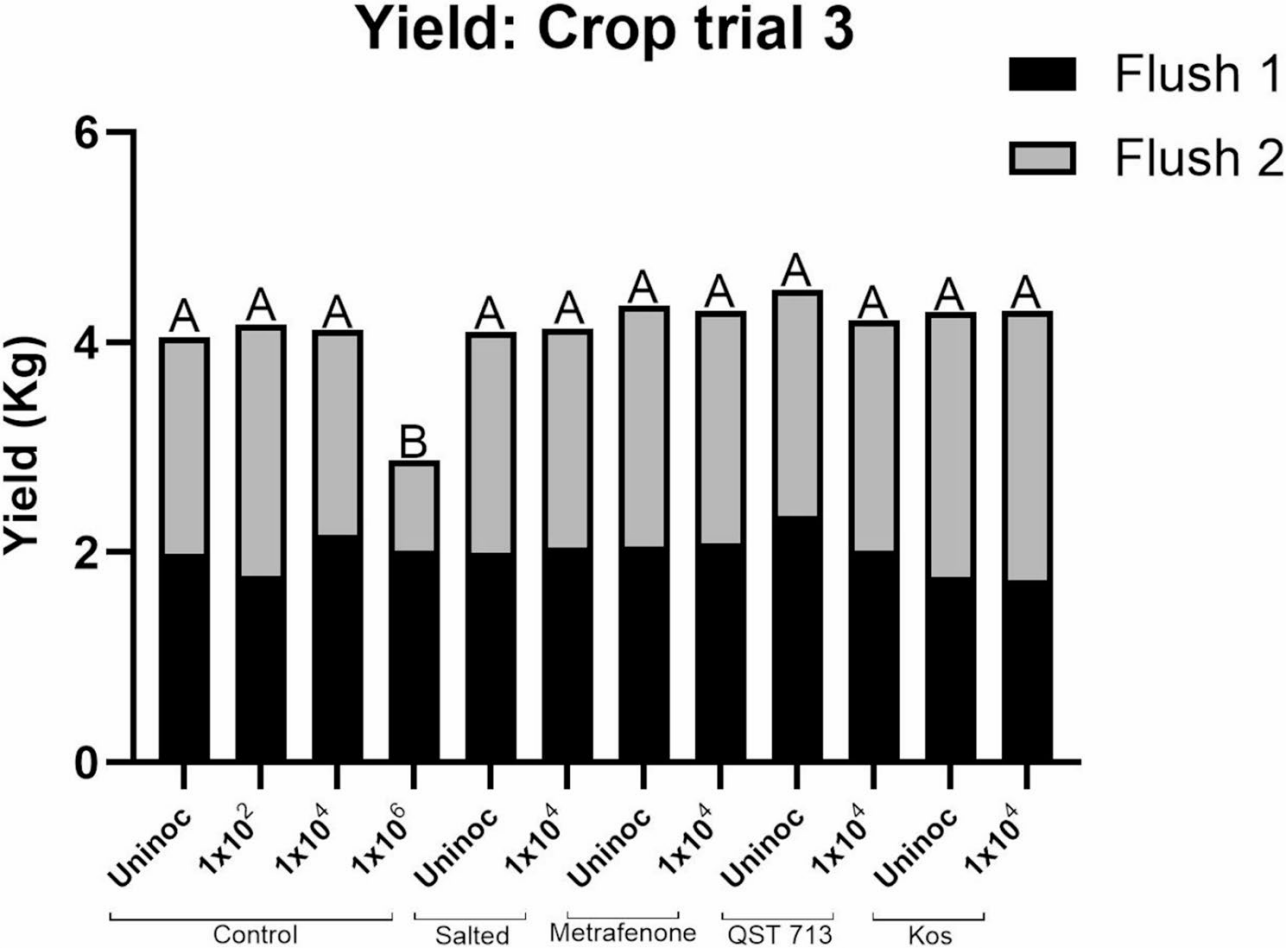
Average healthy yield of *A. bisporus* over two flushes following treatment with salting, the fungicide metrafenone, or the BCAs QST 713 or Kos, followed by inoculation with *L. fungicola* 1722 at inoculation rate of 1 × 10^4^ conidia m^−2^. Untreated control plots were inoculated with either 1 × 10^6^, 1 × 10^4^ or 1 × 10^2^ conidia m^−2^ Data analysed by ANOVA, *n* = 6. Means sharing the same letter are not significantly different at *P* < 0.05 by Tukeys pairwise comparisons test

This crop was not taken into a third flush due to the development of disease in uninoculated plots at the beginning of flush 3, likely due to cross contamination from the extremely high number of bubble mushrooms on the 1 × 10^6^ conidia m^−2^ plots. The average yield of healthy mushrooms collected over two flushes during crop trial 3 can be seen in Fig. 7. The average yield ranged from 1.7 to 2.3 kg plot^−1^ for flush 1 and 0.85 to 2.6 kg plot^−1^ for flush 2. Over the course of this crop trial, the only plots that had a statistically significant reduction in their yield compared to the uninoculated control plots were the control plots inoculated at 1 × 10^6^ conidia m^−2^, confirming earlier results. Total yield over two flushes for the uninoculated controls across all treatments ranged from 2.9 to 4.5 kg plot^−1^. The average yield of each treatment harvested at the end of trial 3 can be found in Table S5.

#### Dry bubble disease

**Fig. 8:**
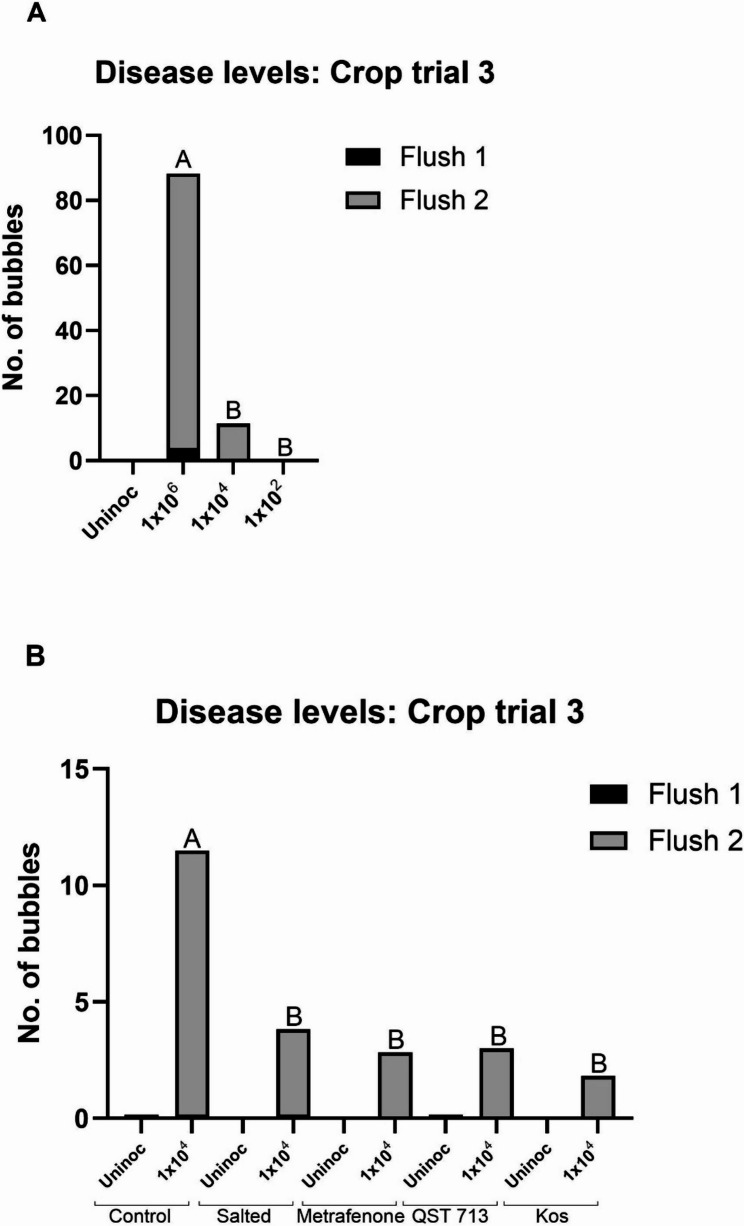
**A** Average number of bubbles recorded at the end of crop trial 3 for control plots inoculated with either 1 × 10^6^ conidia m^−2^, 1 × 10^4^ conidia m^−2^ or 1 × 10^2^ conidia m^−2^ *L. fungicola* 1722. **B** Average number of bubbles recorded at the end of crop trial 3 for plots salted, treated fungicide metrafenone or the BCAs QST 713 or Kos, followed by inoculation with 1 × 10^4^ conidia m^−2^ *L. fungicola* 1722. Data analysed by ANOVA, *n* = 6. Means sharing the same letter are not significantly different at *P* < 0.05 by Tukeys pairwise comparisons test

A few bubble mushrooms were present at the end of flush 1, with the majority being on the control plots inoculated at the 1 × 10^6^ conidia m^−2^ rate. Very few bubble mushrooms were present in flush 1 on any treatment inoculated at the 1 × 10^4^ conidia m^−2^ rate (Table S6). At the end of flush 2, the average number of bubbles in the control plots inoculated at the 1 × 10^6^ conidia m^−2^ rate was 88, which was significantly higher than disease development in either 1 × 10^4^ conidia m^−2^ or 1 × 10^2^ conidia m^−2^ inoculated plots, and which had an average of 11 and 0 bubble mushrooms respectively (Fig. 8 A). This confirmed the results in crop trial (1) There were significantly more bubble mushrooms developing on control plots inoculated with 1 × 10^4^ conidia m^−2^ *L. fungicola* compared to the salted, metrafenone, *B. velezensis* QST 713 and Kos plots inoculated at the same concentration (*p* < 0.05) (Fig. 8B), and this also confirmed the results in crop trial (2) Disease development data for crop trial 3 is summarised in Table S6.

## Discussion

During this work, the treatment of dry bubble disease with fungicide and biocontrol treatments was investigated. The growth of *L. fungicola*, isolate 620 and 1722 in liquid cultures was significantly reduced when treated with the fungicides prochloraz and metrafenone in vitro with concentrations as low as 1 mg×kg^−1^. It was also shown that the growth of both isolates in the presence of 500 mg×kg^−1^ metrafenone was higher than the growth recorded with lower metrafenone concentrations. This suggest that there is a threshold where inhibition of *Lecanicillium* isolates begins to decline. The development of fungicide resistance in pathogenic isolates is a major concern for mushroom growers, particularly considering the limited availability of fungicide treatments. Prochloraz is classified as a demethylation-inhibitor (DMI) fungicide which reduces fungal growth through inhibition of fungal sterol biosynthesis [39]. The development of prochloraz resistance has been linked with target site modifications to the CYP51 gene family, as well as the involvement of efflux transporters that reduce intracellular fungicide concentrations [40, 41]. However, up until its recent loss of approval, prochloraz still provided good control of cobweb isolates during crop trial experiments [21]. Metrafenone belongs to the benzophenone class of fungicides, its antifungal effects are believed to be due to the disruption of fungal hyphal morphogenesis and cell polarity [42]. The resistance mechanisms towards metrafenone are currently under investigation [43]. The results of the in vitro experiment indicated that both fungicides significantly reduced the growth of isolates 620 and 1722, suggesting that resistance mechanisms have not yet developed in these strains. However, continued monitoring will be essential to detect potential emergence of fungicide resistance in the future. Only isolate 1722 was included in the crop trials. Previous work has shown that the culture filtrate from *B. velezensis* Kos and the biocontrol product Serenade^(R)^, which contains *B. velezensis* QST 713 was also able to significantly reduce the growth of *L. fungicola*, isolate 1722 in vitro [36].

One of the aims of this work was to determine an inoculation rate which would reflect realistic dry bubble disease conditions on mushroom farms. It has been shown that different inoculation levels used during *Trichoderma aggressivum* (green mould disease) crop trial experiments correlates to yield loss and disease symptom severity [44]. During this work, in both crop trial 1 and crop trial 3, inoculation with a rate of *L. fungicola* 1 × 10^6^ conidia m^−2^ in untreated control plots significantly increased bubble development compared to the uninoculated controls (*p* < 0.05). In crop trial 1, there was no significant difference between the bubble development in the untreated control plots and prochloraz, *B. velezensis* QST 713 or Kos treated plots inoculated at a rate of 1 × 10^6^ conidia m^−2^. Bubble symptom also began to appear during the first flush of mushrooms. Growers generally report dry bubble disease occurring mid-crop, from about flush 2 onwards, which is supported by the results of a farm survey conducted between 2008 and 2010 [45]. In crop trial 3, bubble mushrooms also developed extremely quickly and at a high rate when plots were inoculated at 1 × 10^6^ conidia m^−2^. The yield of all plots given the 1 × 10^6^ conidia m^−2^ inoculation, regardless of treatment application was statistically reduced compared to the uninoculated control (*p* < 0.05) in both crop trial 1 and 3. These results suggested that *L. fungicola* 1722 at an experimental inoculation rate of 1 × 10^6^ conidia m^2^ was too high to be controlled by the fungicide, prochloraz or the biocontrol treatments examined in this work, although prochloraz did reduce the number of bubbles in the first flush. The results for prochloraz were surprising as this fungicide has been generally reported as effective against dry bubble disease [33, 37, 46, 47]. Prochloraz was most effective during the first flush but showed little to no efficacy during flush 2 and 3. The reduced efficacy in later flushes may be linked to changes in the post-harvest interval for prochloraz, which was increased to 10 days in the mid-2010s. This resulted in label revisions, reducing applications to a single 1 g/m^2^ application at least 10 days before harvesting. As a result, up until the loss of approval in 2023, most growers were applying a single application of prochloraz during case-run. The use of a single application during case run also means that prochloraz concentration in the casing layer would decrease over time, leading to a reduction of effective concentration to reduce disease symptoms [37, 48]. An infection does of 1 × 10^6^ conidia m^−2^ could represent extremely high disease levels that may not normally be seen on a farm with good disease monitoring and treatment practices in place. Prochloraz may have been expected to have better efficacy at this rate, but it has been suspected that this rate is too high for biocontrol treatments to suppress. Prochloraz is a popular fungicide treatment for several field crops. It can effectively inhibit pathogen growth by inhibition of the cytochrome P450-dependent 14a-demethylase but has been linked with high levels of toxicity [39]. The effectiveness of prochloraz against dry bubble disease has been known for many decades [37, 46, 49] and has been a popular treatment for growers to control disease. Stanojević *et al*., (2019) [33] did find that an inoculation of 1 × 10^6^ conidia ml^−1^ *L. fungicola* strain Sa_2_V_6_ isolated in Serbia, could be controlled by prochloraz. However, there have been reports of reduced in vitro sensitivity of *L. fungicola* isolates to prochloraz [50] and results of the work presented here suggest high inoculations of *L. fungicola* strain 1722 may be less sensitive to prochloraz. Regardless, the use of prochloraz on mushroom crops is no longer approved within the EU [24].

The lower inoculation levels (1 × 10^4^ conidia m^−2^ and 1 × 10^2^ conidia m^−2^) were expected to be more representative of disease pressure present on mushroom farms. There was also no significant difference in disease levels between the lower inoculation rates in control and treatment plots during crop trial 1. It was noted that bubble development was quite inconsistent between replicates plots. Extreme care was taken to salt bubbles to avoid cross contamination between plots during crop trial 1 and any bubble that did appear was salted immediately after identification. It is possible that the diligent salting of bubbles in the lower inoculated control plots was sufficient to prevent major bubble disease outbreak. It is also interesting to note that there was large bubble outbreak in the 1 × 10^6^ conidia m^−2^ inoculated plots during flush 2, which appeared to be suppressed by flush 3, after salting was carried out in crop trial 1. The lack of development of mushroom bubbles in the third flush of the untreated inoculated control was unusual as again, the literature shows that disease usually develops rapidly once a crop is infected [12]. At this point it was apparent that the salting procedure, used to minimise disease spread was actually very effective at preventing disease development. The disease levels in the untreated 1 × 10^4^ and 1 × 10^2^ conidia m^−2^ control plots may not have been representative of untreated disease progression, as the salt prevented *Lecanicillium* conidia from spreading within the plot. This could explain the inconsistencies in disease development on these plots.

To confirm this, we performed a second replicate trial with the two lower inoculation rates (1 × 10^4^ conidia m^−2^ and 1 × 10^2^ conidia m^−2^). In this trial we included an unsalted control treatment as well as a separate salting treatment which was salted as in trial 1. Bubbles were left to develop without any interference during the flush in control, fungicide, and *B. velezensis* treated plots. During this second trial we found once again that disease was mostly absent from plots inoculated with 1722 1 × 10^2^ conidia m^−2^. This would suggest that this inoculation rate is too low for dry bubble disease to develop in an experimental setting. The scarce bubble that did develop from these plots, only appeared during the third flush, which would suggest that dry bubble in the third flush is likely to reflect low disease pressure on the farm. This was replicated in crop trial 3 as there was also no bubble development for the 1 × 10^2^ conidia m^−2^ plots.

In crop trial 2, there was development of dry bubble disease in the plots inoculated with *L. fungicola* 1722 at a rate of 1 × 10^4^ conidia m^−2^ which was first identified on these plots during flush 2. By the end of crop trial 2, there were significantly higher bubble levels in the infected control plots compared to the salted, fungicide metrafenone and biocontrol *B. velezensis* QST 713 and Kos treated plots. This result was replicated in the third crop trial where once again, salting, metrafenone, QST 713 and Kos treatment significantly reduced bubble development on plots inoculated with *L. fungicola* at a rate of 1 × 10^4^ conidia m^−2^.

It was found that there were significantly higher levels of bubble development on the control unsalted plots compared to the salted plots in two replicate crop trials. This is understandable as bubble disease is spread by watersplash, which would have happened in the unsalted treatments after the second flush. This can be seen also in the data from crop trial 1 (1 × 10^6^ conidia m^−2^ plots), where very few bubbles developed in the third flush as all the bubbles in the second flush had been salted. During crop trial 3, when no salt was applied to plots inoculated at 1 × 10^6^ conidia m^−2^, the average number of bubble mushrooms rose to 88 compared to an average of 31 in crop trial 1. These results confirm that carefully salting bubbles is effective as a treatment for bubble without any additional preventative treatment and is a useful and worthwhile technique for growers to employ on their farm.

The fungicide metrafenone preformed the best out of all treatments included in crop trial 2 and 3 with an efficacy value of 96% at the end of the three flushes, demonstrating that the only remaining fungicide for mushroom disease is effective against dry bubble. Due to the lack of any alternative fungicide, it is likely that the development of metrafenone resistance strains will be difficult to avoid. Previous research has demonstrated how metrafenone treatment was effective for the treatment of cobweb diseases during crop trial experiments [51]. However, during recent crop trials carried out, metrafenone tolerant isolates of *Cladobotryum* were identified [21].

Fortunately, biocontrol strains also performed well against dry bubble disease at this moderate inoculation rate of 1 × 10^4^ conidia m^−2^. *B. velezensis* QST 713 had the second highest efficacy of 85%, followed by *B. velezensis* Kos with an efficacy of 73%. Stanojević *et* *al*.,(2019) [33] also investigated the use of *B. velezensis* QST 713 to control dry bubble disease and found that although it did not perform as well as the prochloraz fungicide treatment, it did show a level of protection against a high inoculation rate of *L. fungicola*. However, Navarro *et al*.,(2023) [52] reported that *B. velezensis* QST 713 had limited efficacy in controlling wet bubble disease, despite infecting crops with relatively low inoculum concentrations (10^3^ CFU m^−2^). We have previously shown that *B. velezensis* QST 713 and Kos can inhibit the growth of the *L. fungicola* pathogen in vitro. Proteomic analysis revealed that in response to the CF of the two strains, *L. fungicola* significantly reduces growth activities and increases activities involved with a stress response [36]. Several lytic enzymes, including subtilisin were also identified in the inhibitory CF fraction of *B. velezensis* Kos, which may contribute to the antagonistic potential of this strain [35]. Genomic clusters responsible for the biosynthesis of antimicrobial secondary metabolite genes have also been identified in this strain. These genes encode for surfactin, subtilin, bacillibactin, bacilysin, fengycin, bacillaene and macrolactin [53].

## Conclusions

Using different inoculation levels in crop trials can allow various disease conditions to be tested. It is our recommendation that an inoculation rate of 1 × 10^4^ conidia m^−2^ would represent the optimum experimental inoculation rate of *L. fungicola* to represent a reasonable level of dry bubble disease conditions in an experimental setting.

Biocontrol treatments showed efficacy against *L. fungicola* infection when disease levels were low/moderate. The results from previous in vitro inhibition work [31] and these large-scale crop trials, suggests that there is potential for the use of biocontrol treatments to treat dry bubble disease. Salting and early detection of symptomatic areas on mushroom beds can also significantly prevent disease spread when infection levels were low/moderate.

The future of mushroom disease control will likely need to include several IPM techniques working in combination. Biocontrol agents/treatments struggle to control high disease pressures, therefore, in order to maximise the effects of biocontrol treatment, it will need to be combined with other IPM techniques, such as salting, excellent hygiene, establishment of disease prevention practices and providing training for mushroom pickers to be able to identify disease symptoms early.

## Supplementary Information

Supplementary Material 1.

Supplementary Material 2.

## Data Availability

The authors declare that the data supporting the findings of this study are available within the paper and its supplementary information files. Should any raw data files be needed in another format they are available from the corresponding author upon reasonable request. The results of this manuscript were part of a PhD thesis (Evaluation of the efficacy of fungicide and biocontrol treatments for the control of disease on *Agaricus bisporus* mushroom crops) submitted to Maynooth University in 2024.

## Abbreviations

A.I: Active Ingredient
BCA: Biological Control Agent
EC: European Commission
EU: European Union
IPM: Integrated Pest Management
SUD: Sustainable use of Pesticides Directive
PDA: Potato Dextrose Agar
SDB: Sabouraud Dextrose Broth
CF: Culture Filtrate
NB: Nutrient Broth
CFUs: Colony Forming Units
DI: Disease Incidence
ANOVA: Analysis of Variance
