# Supplementary material for: The control of mushroom pathogen Lecanicillium fungicola with fungicides and Bacillus-based biocontrol treatments during crop trial studies
Source: BMC Microbiol. 2025 Nov 20;25:767. doi: 10.1186/s12866-025-04356-y (PMC12632097; doi:10.1186/s12866-025-04356-y)
Supplement: Supplementary file 1 — Supplementary Material 1. [file 12866_2025_4356_MOESM1_ESM.docx]

Control 1 mg×kg^-1^ 10 mg×kg^-1^ 100 mg×kg^-1^ 500 mg×kg^-1^

**Isolate 620: Metrafenone**


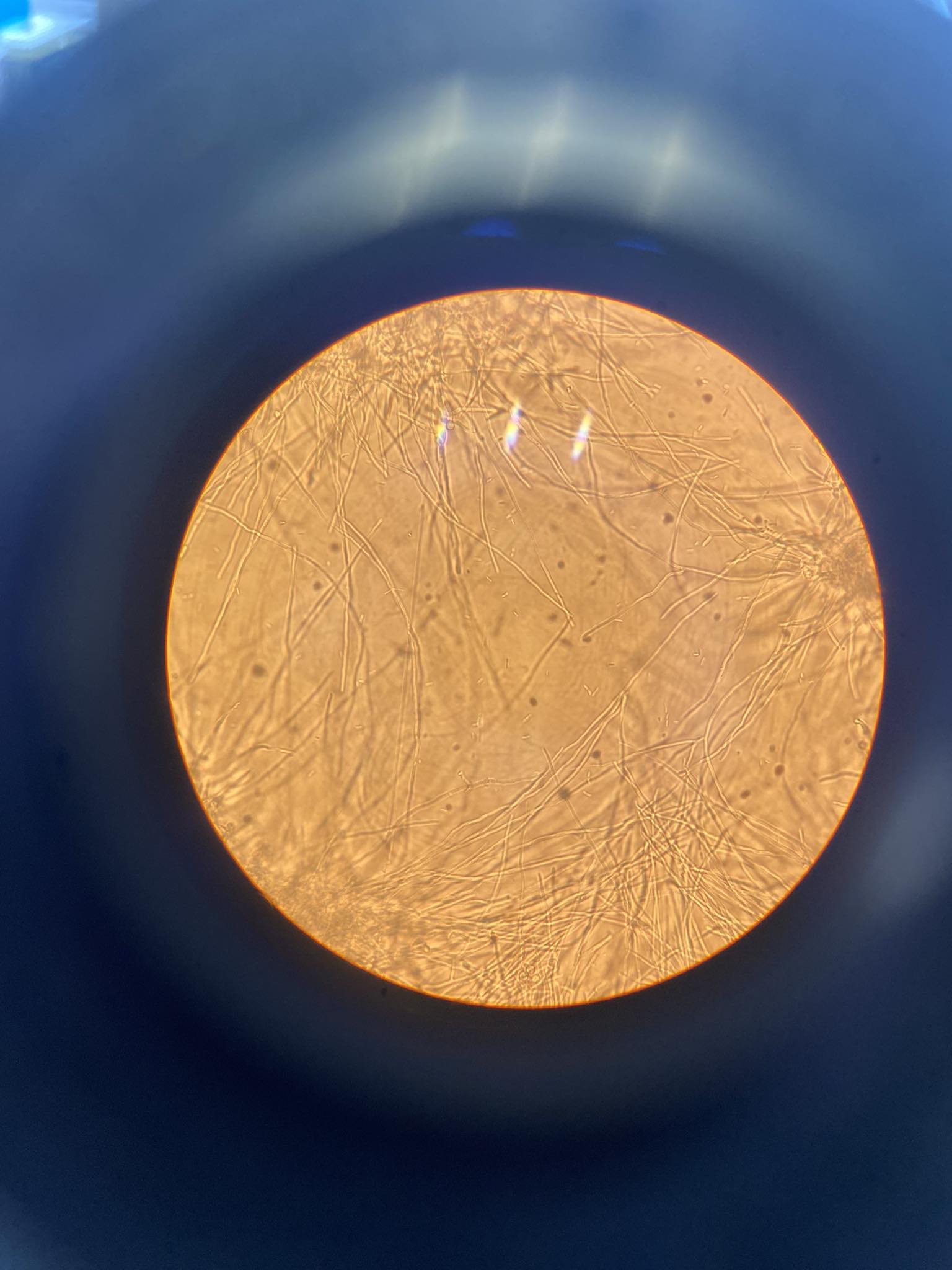

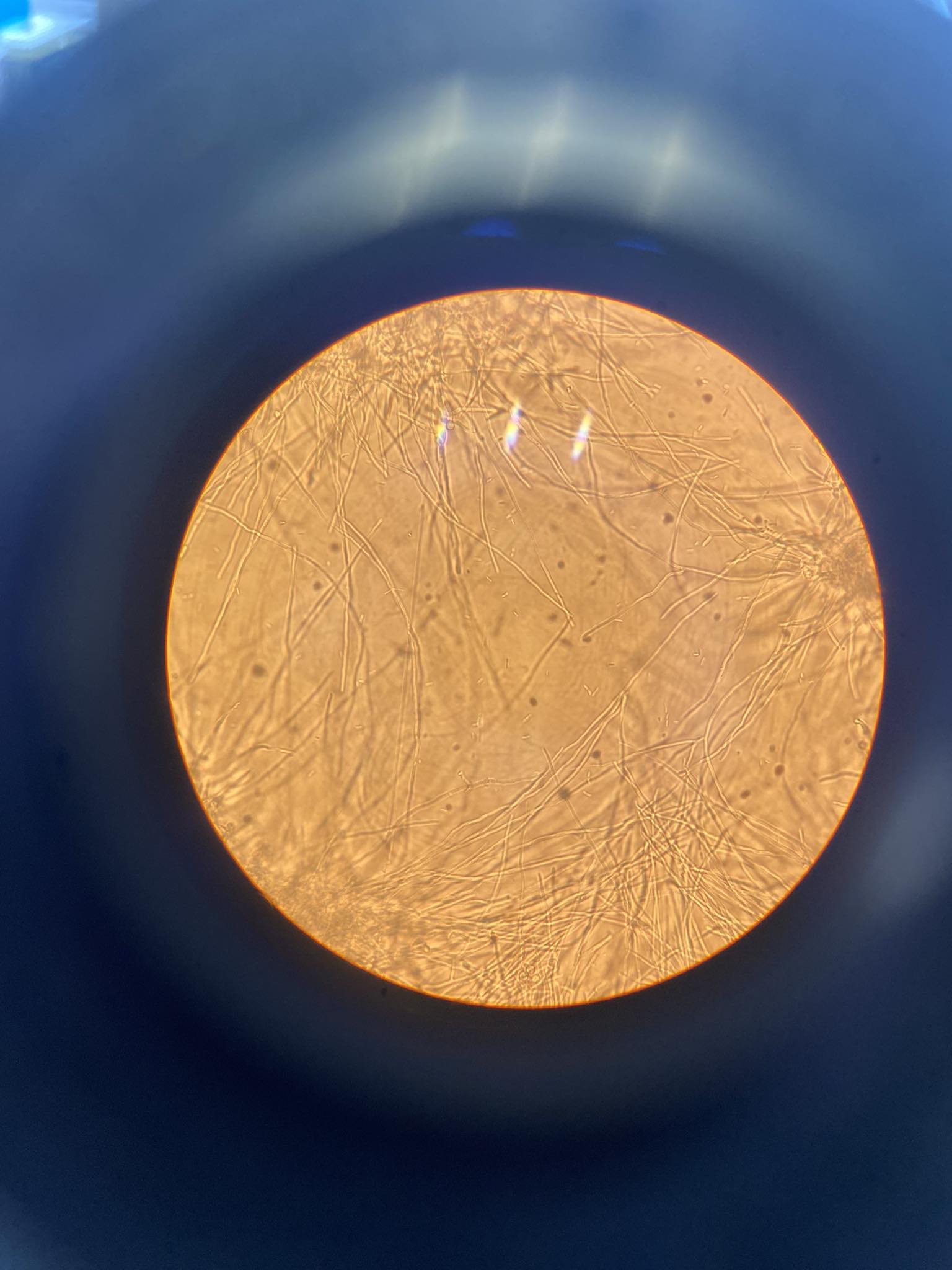

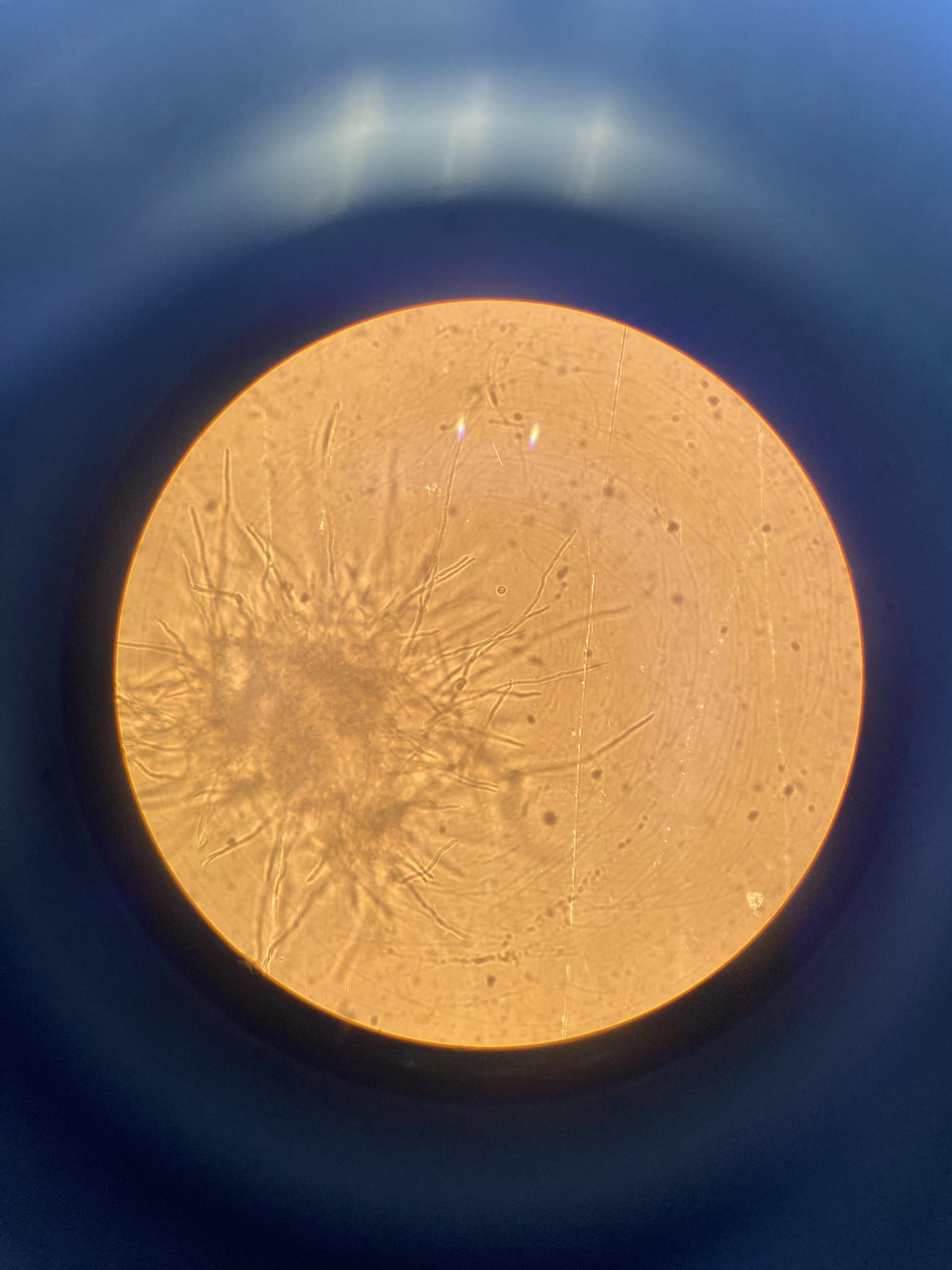

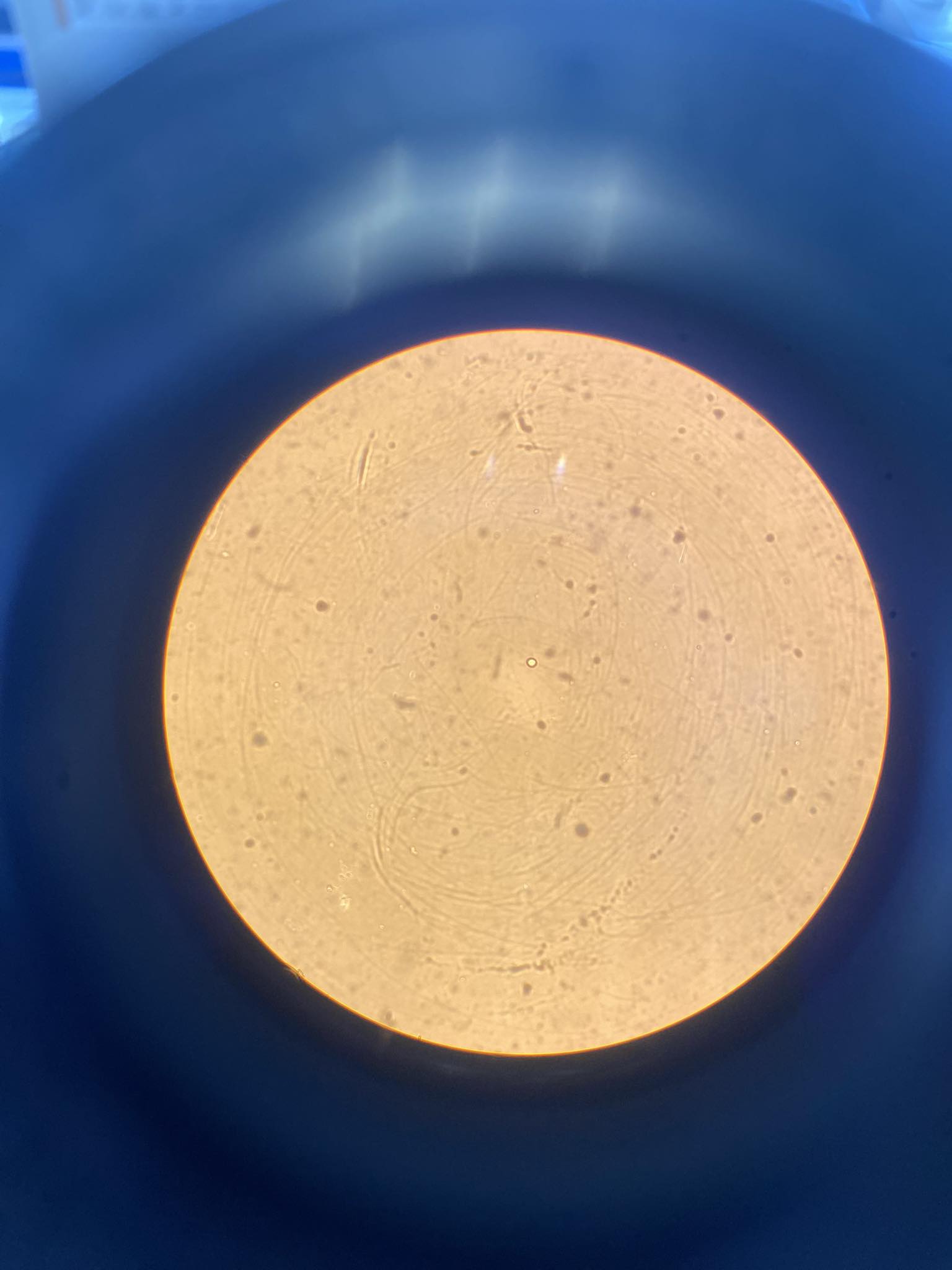

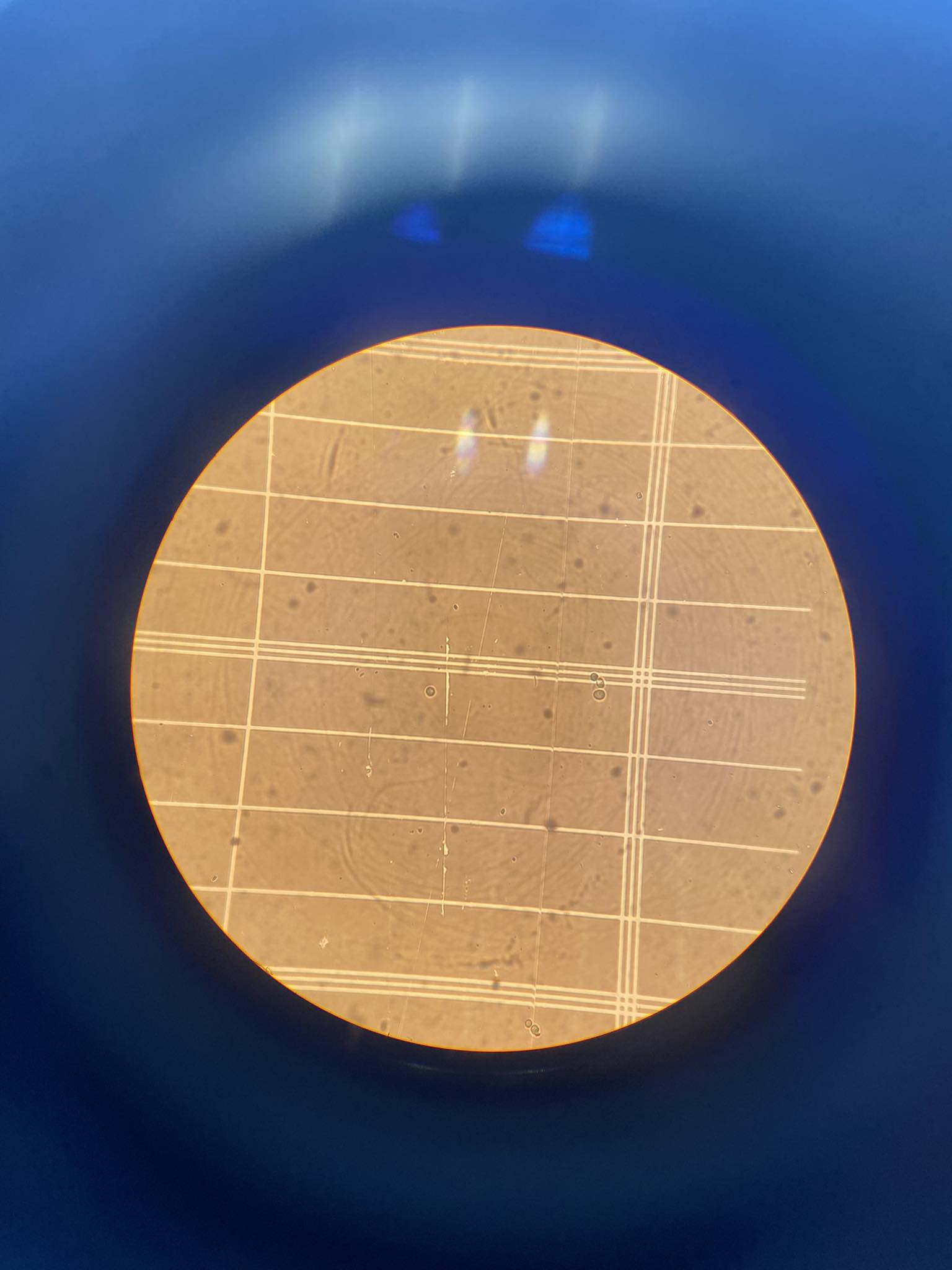

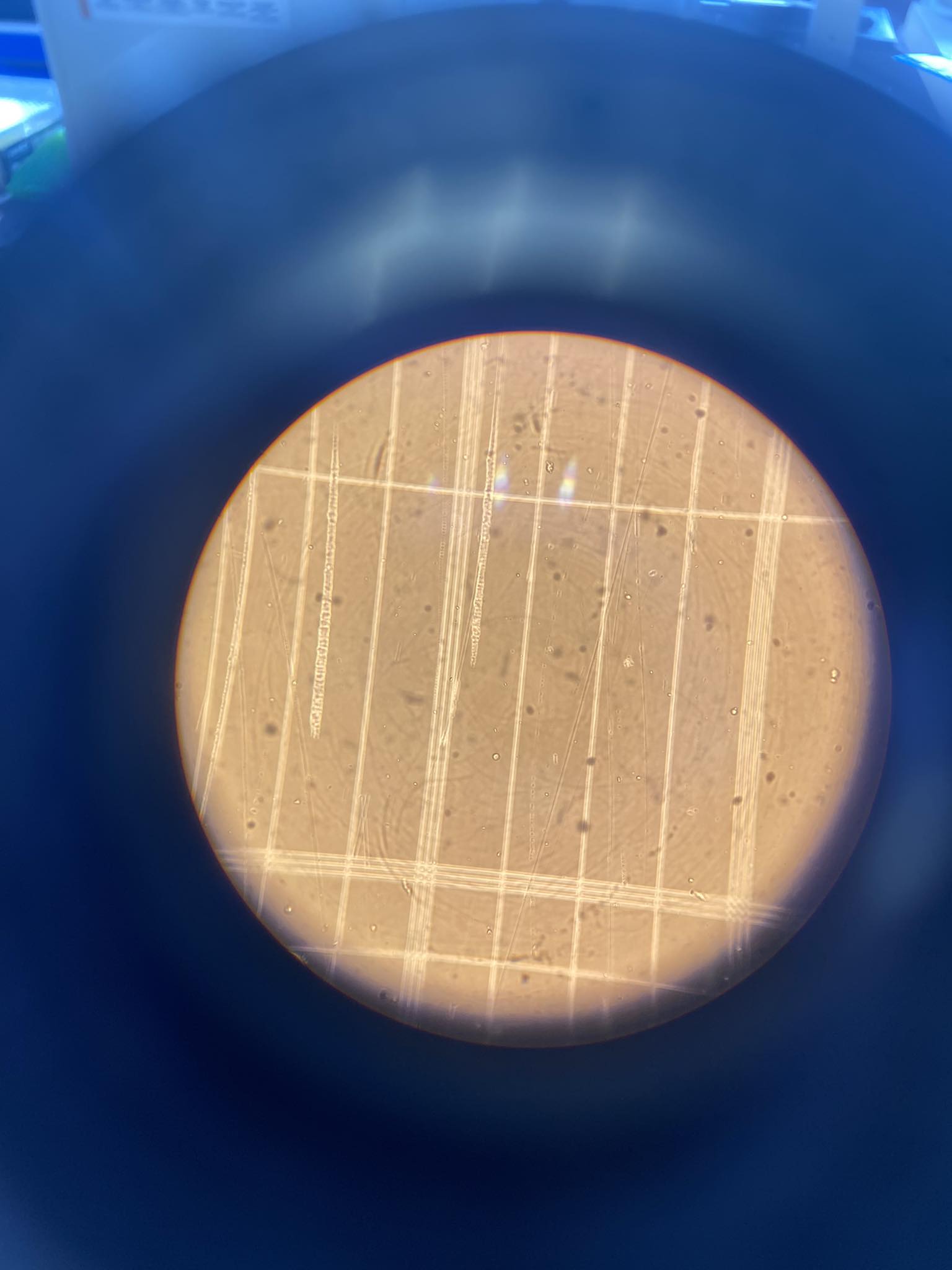

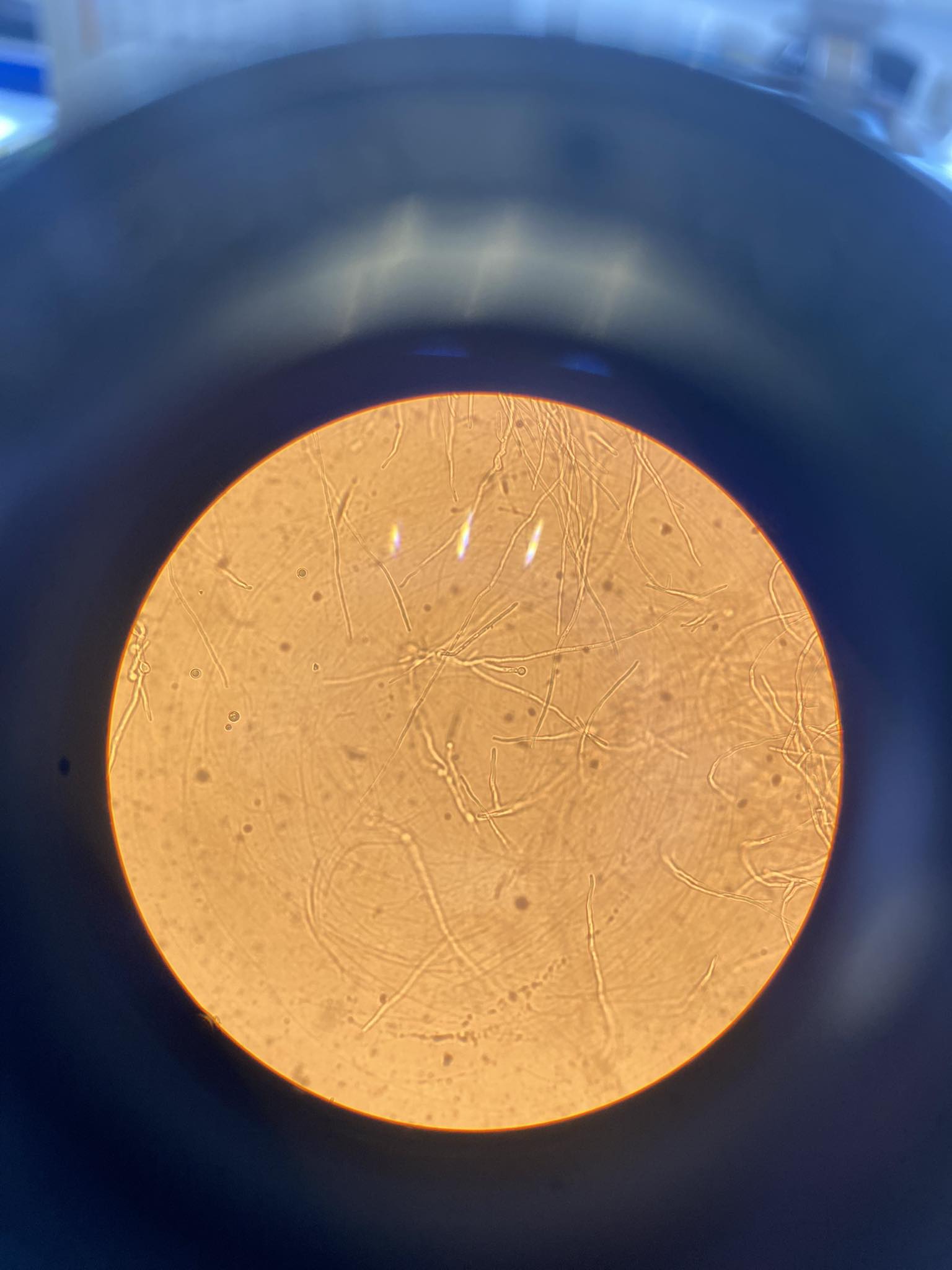

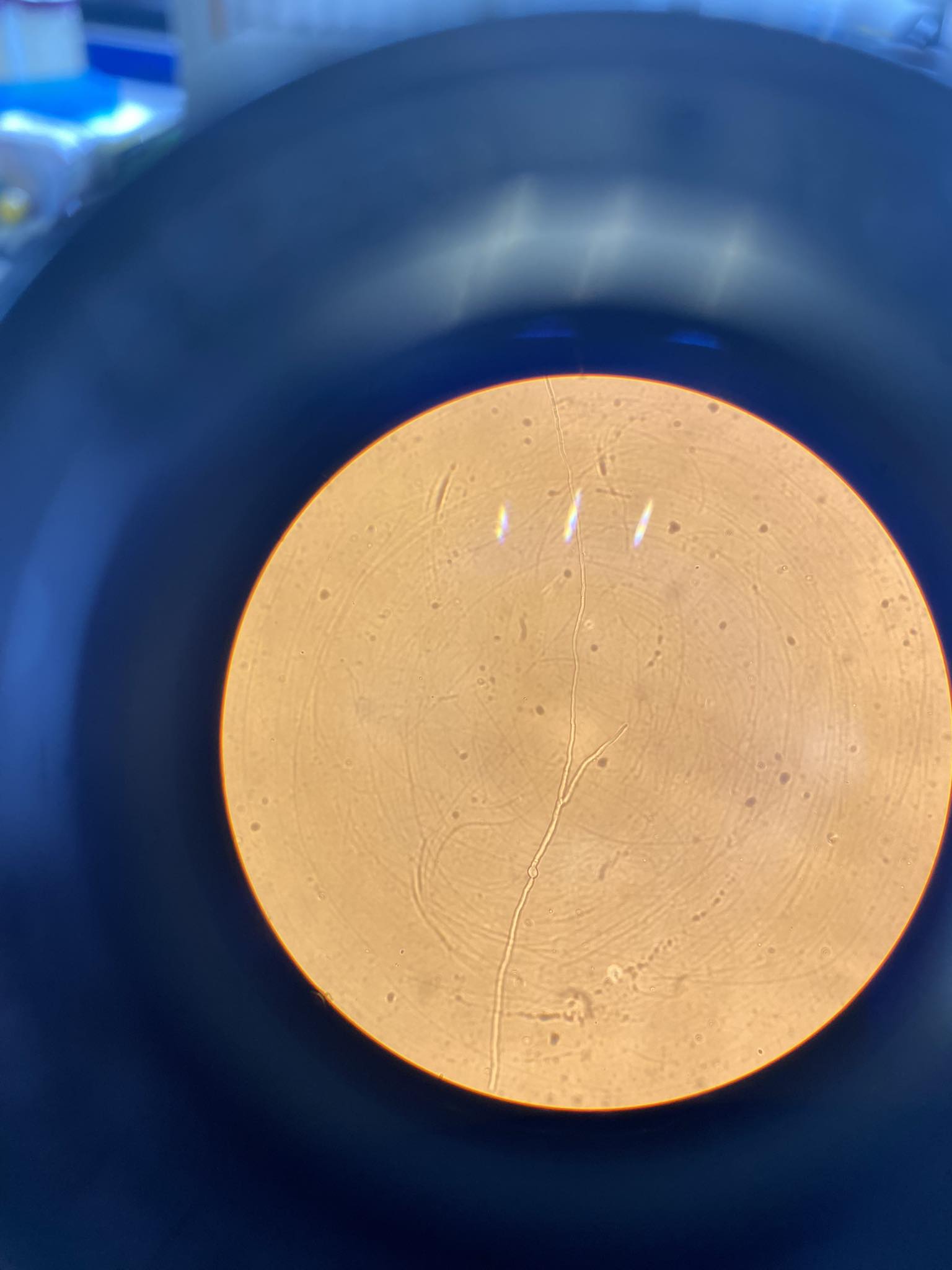

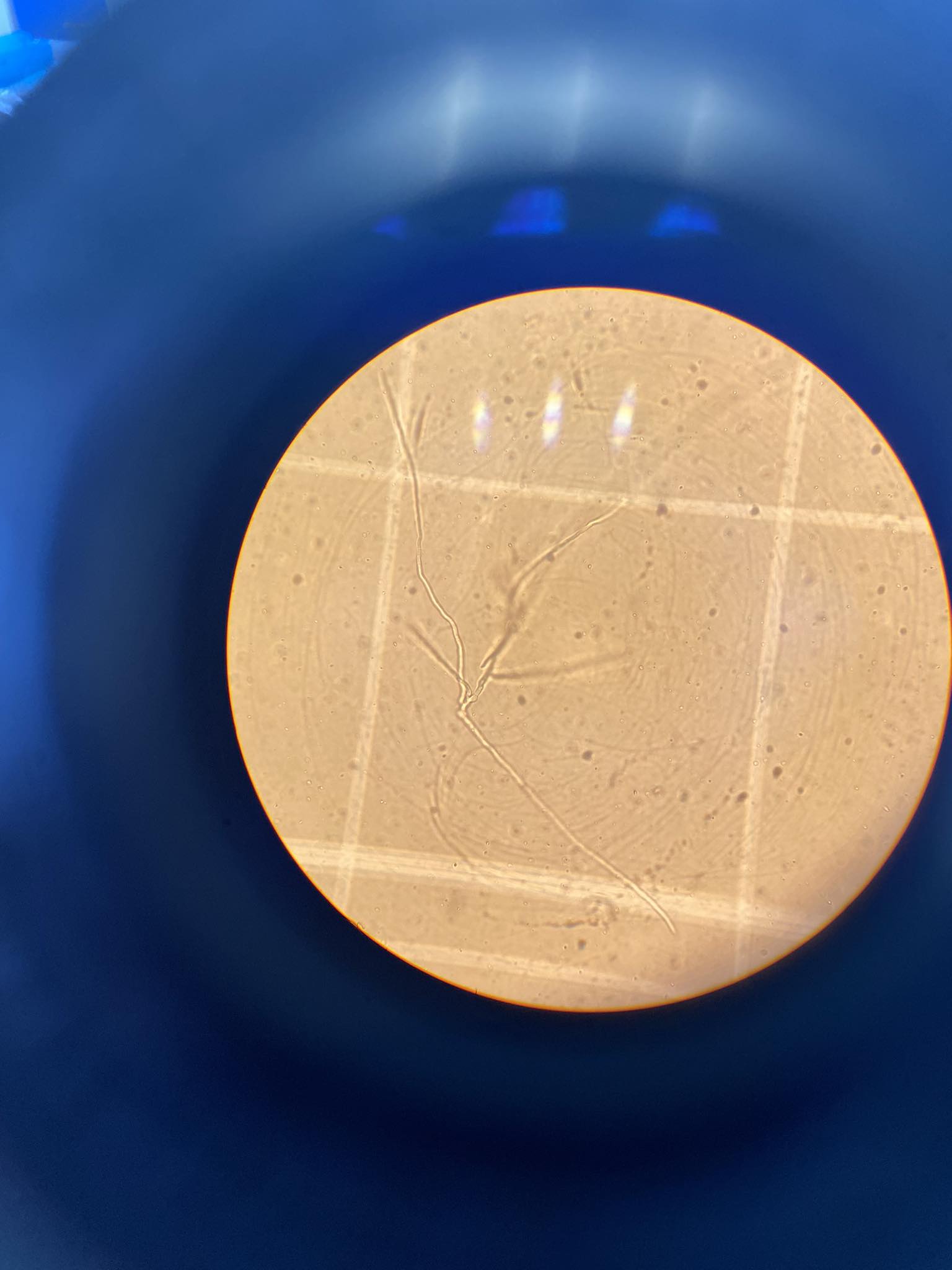

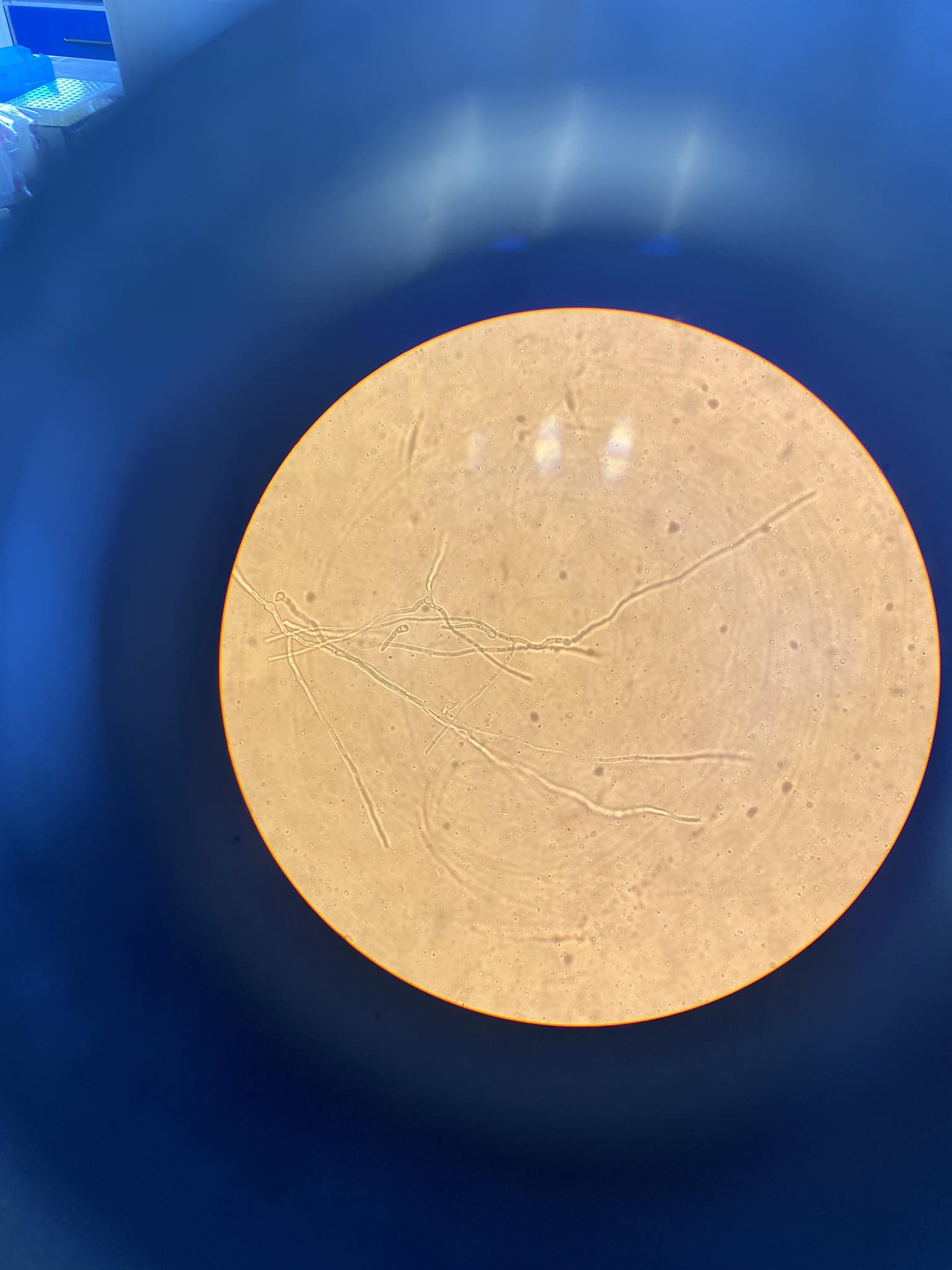


**Isolate 620: Prochloraz**

Control 1 mg×kg^-1^ 10 mg×kg^-1^ 100 mg×kg^-1^ 500 mg×kg^-1^

**Figure S1A:** Investigation of conidiation and hyphal development of *L. fungicola* 620 when treated with fungicides prochloraz or metrafenone at 0, 1, 10, 100 and 500 mg×kg^-1^for 24 hr. Evidence of conidiation and hyphal development was monitored using an Olympus microscope (40X).


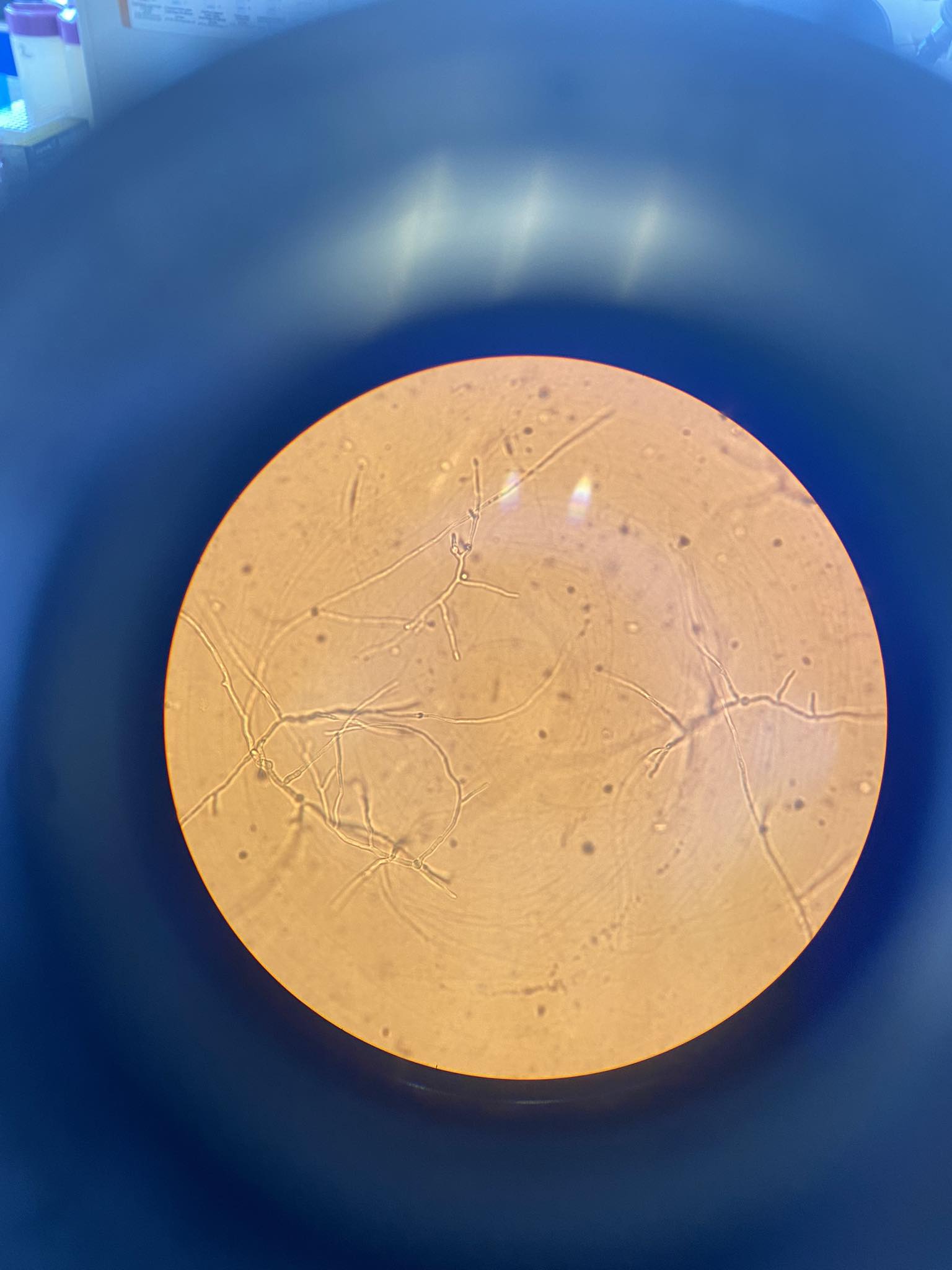

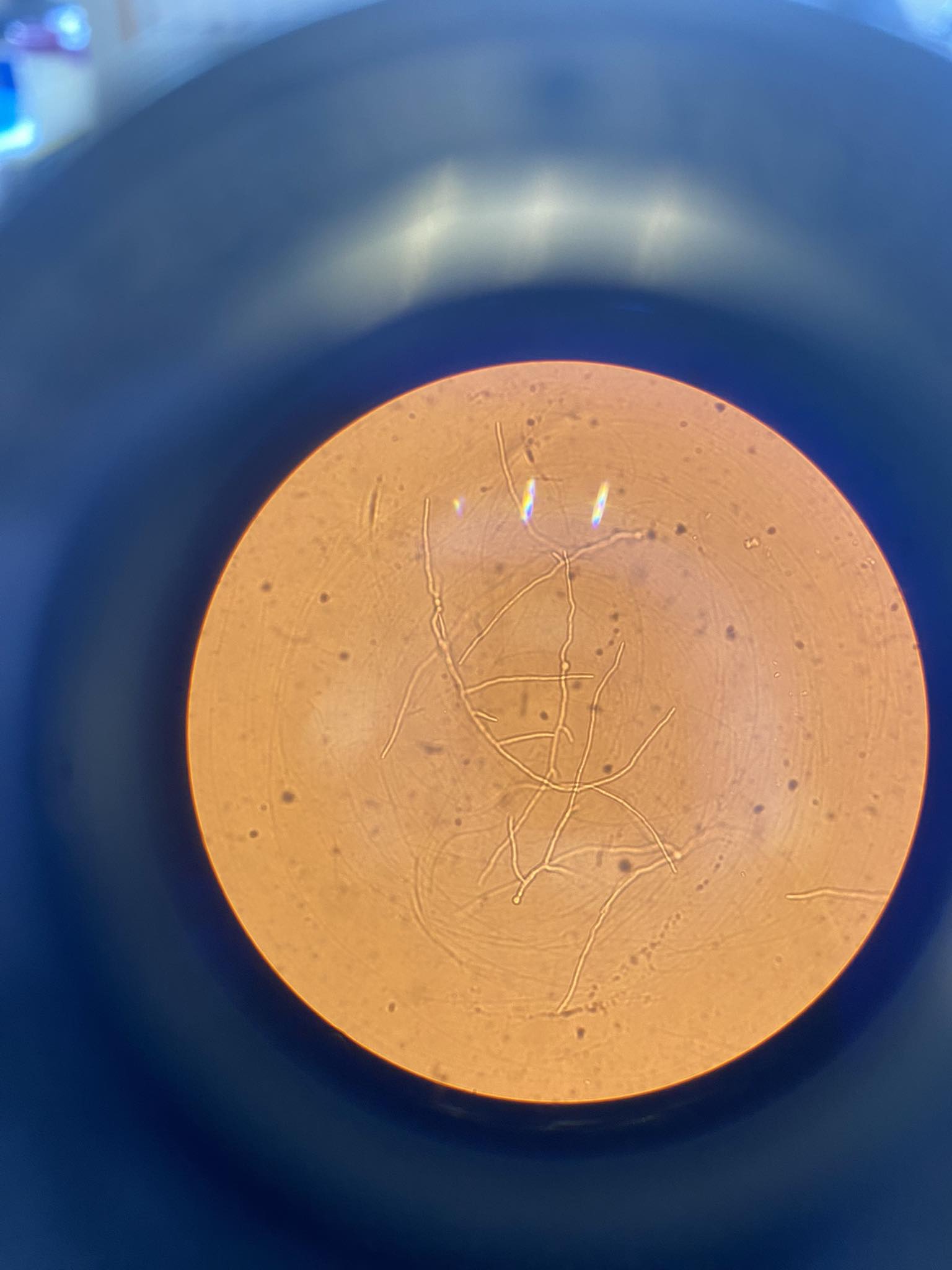

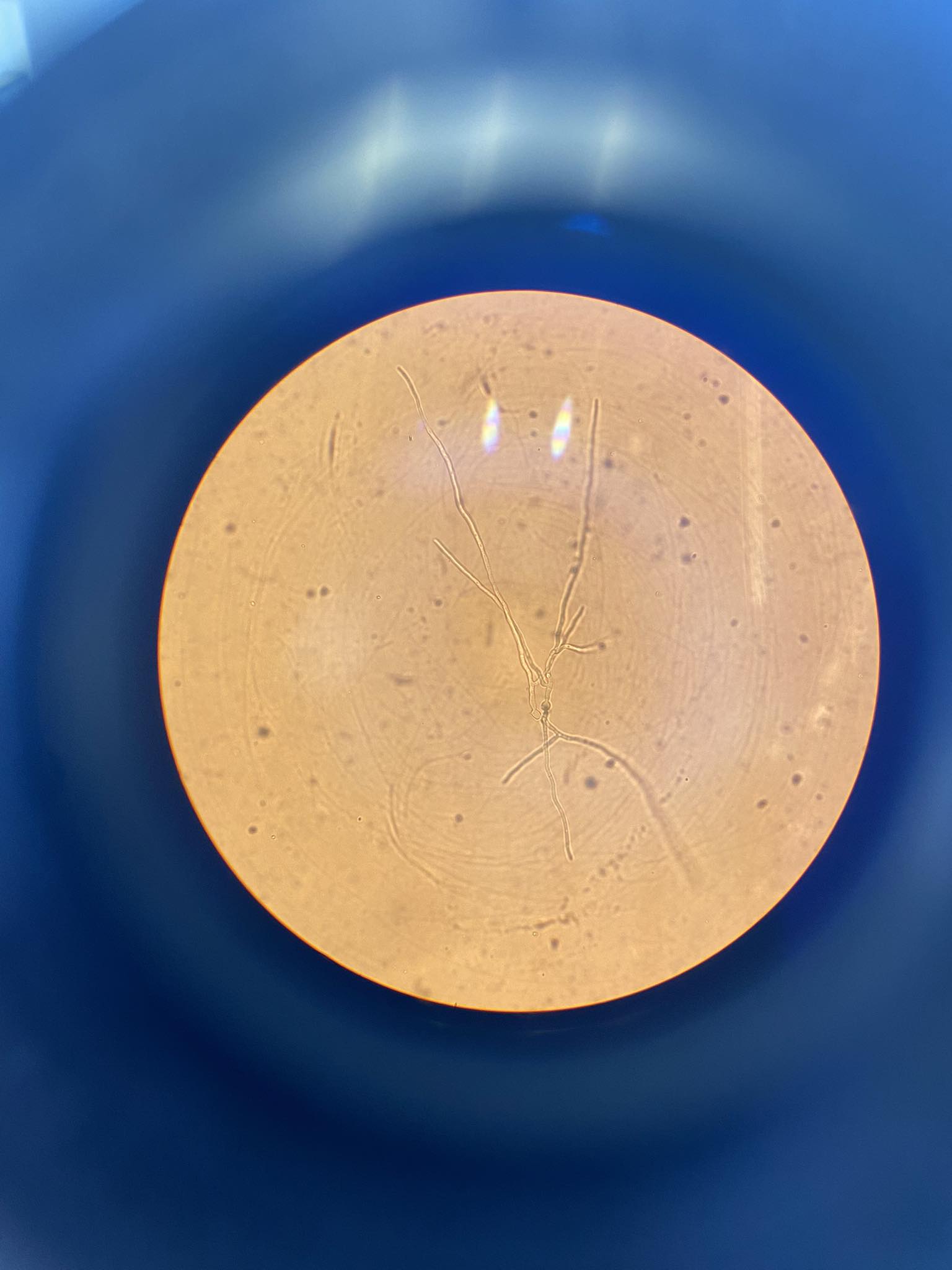

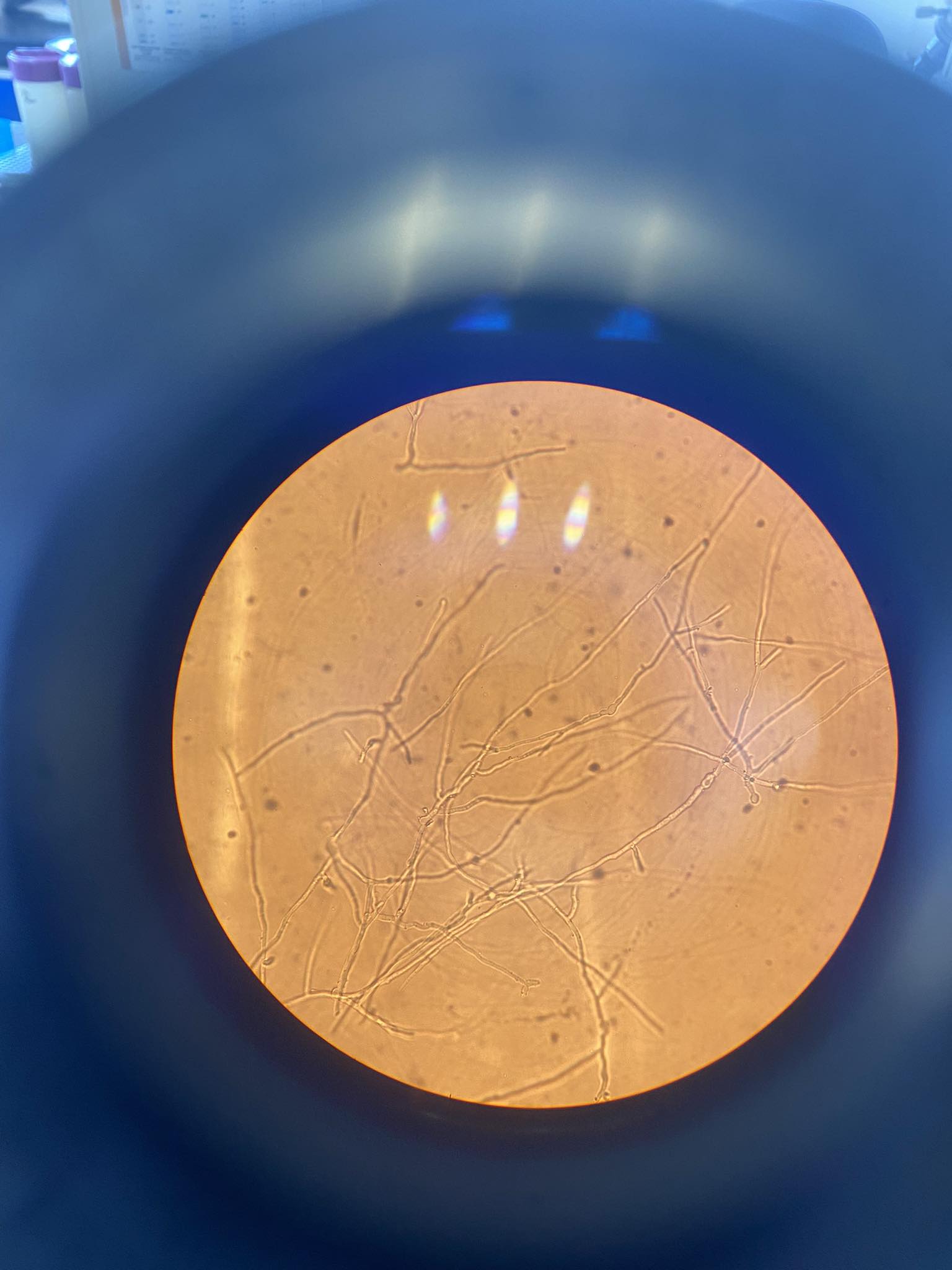

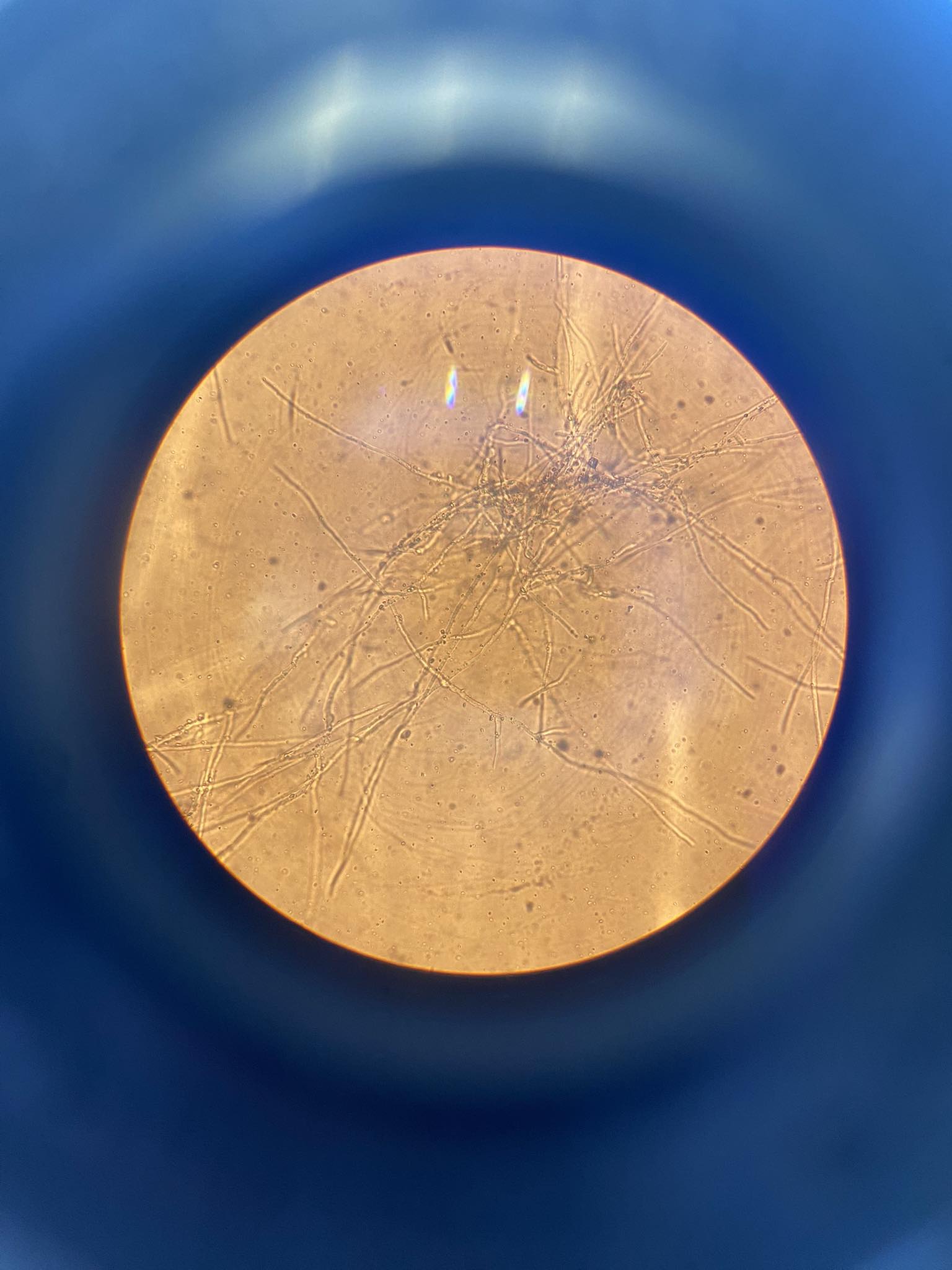


**Isolate 1722: Metrafenone**

**Isolate 1722: Prochloraz**


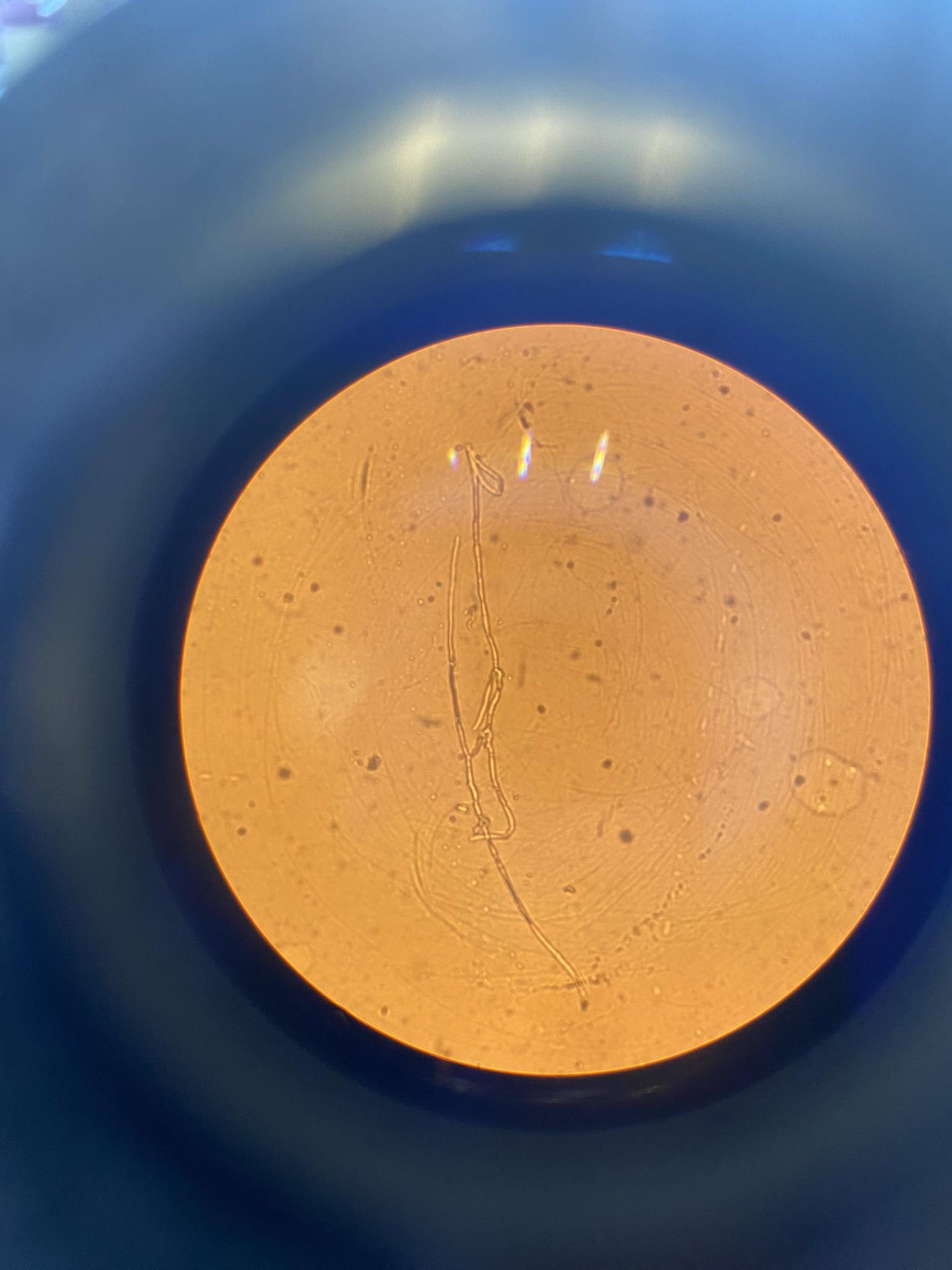

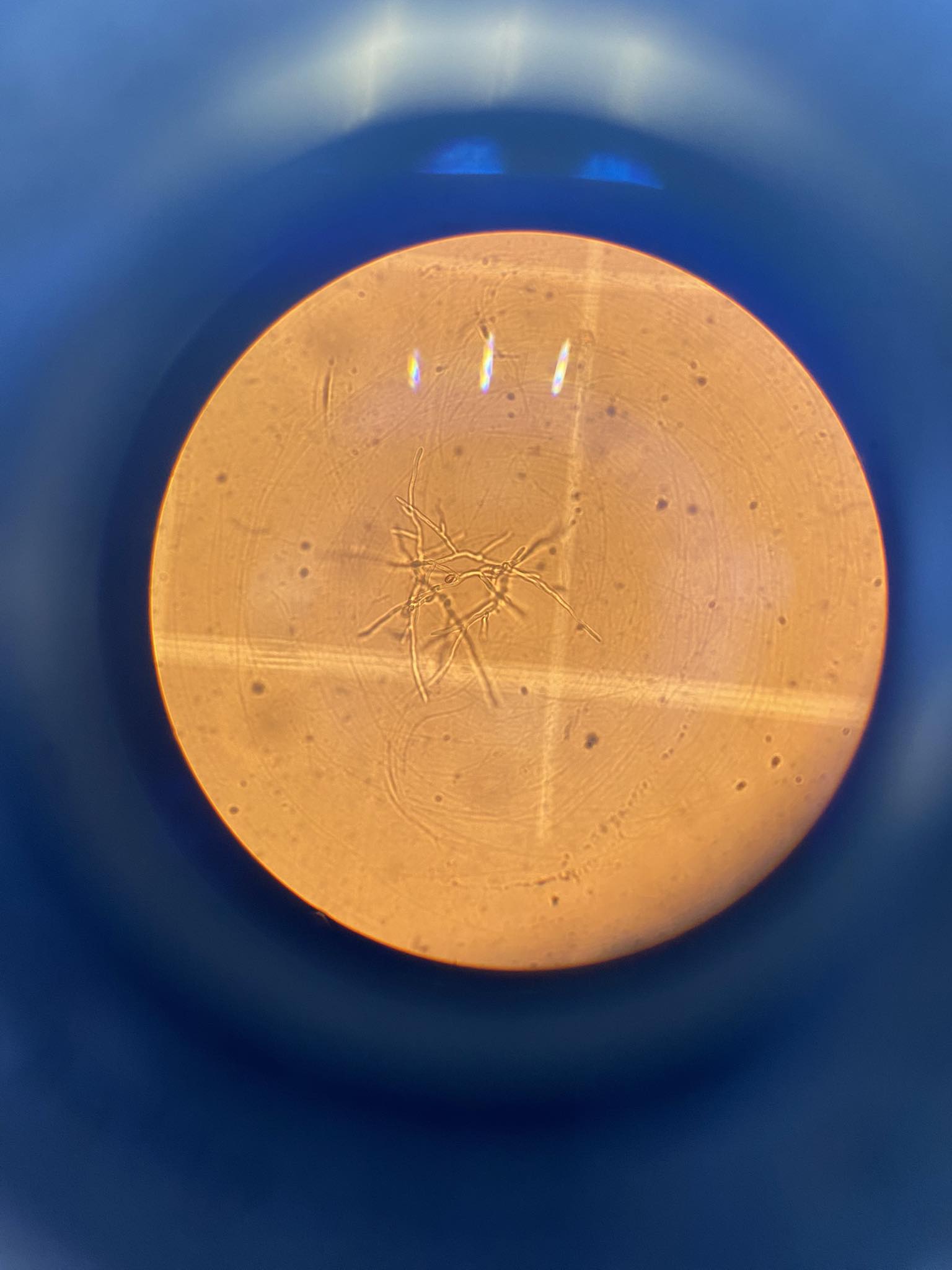

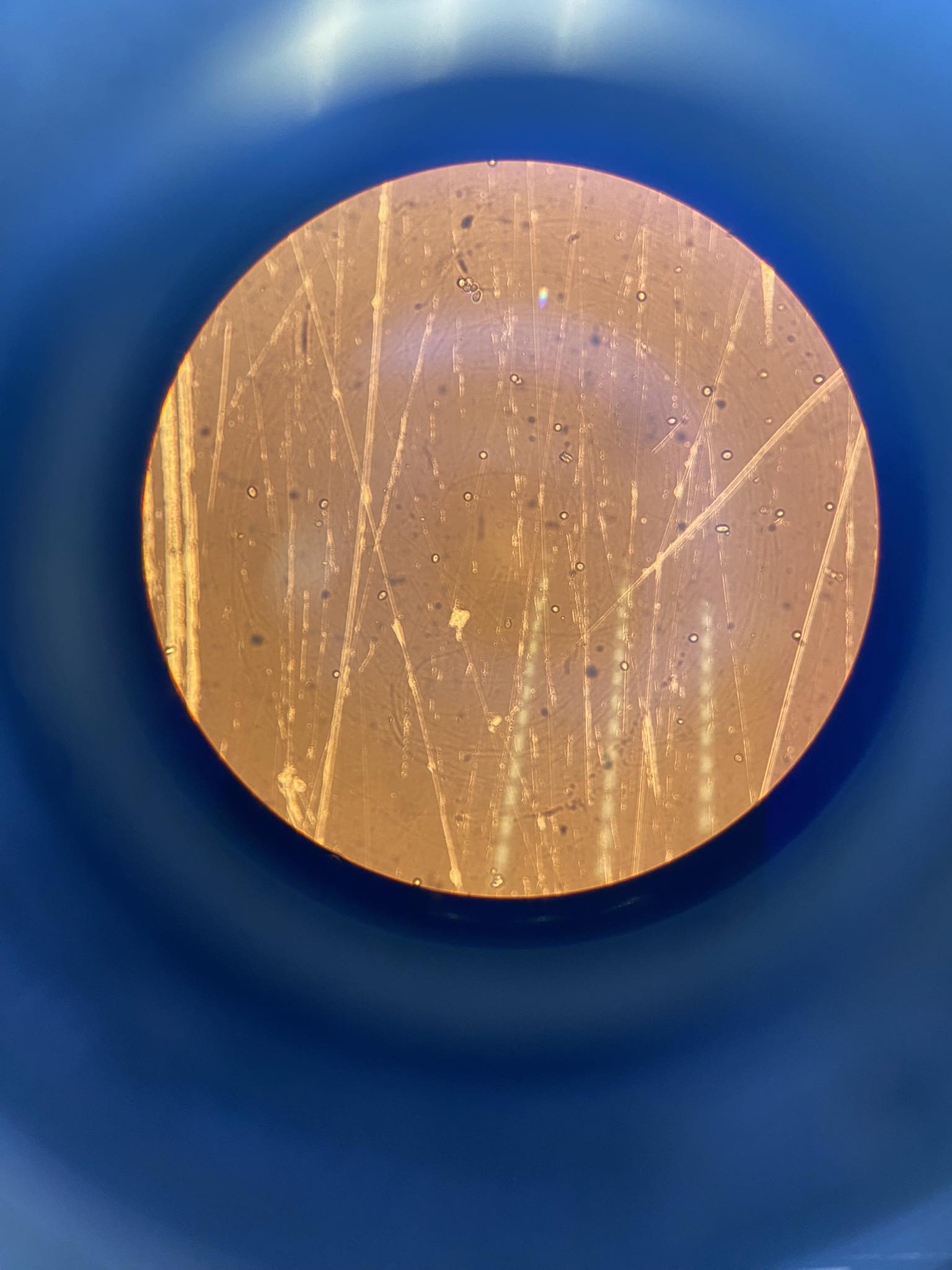

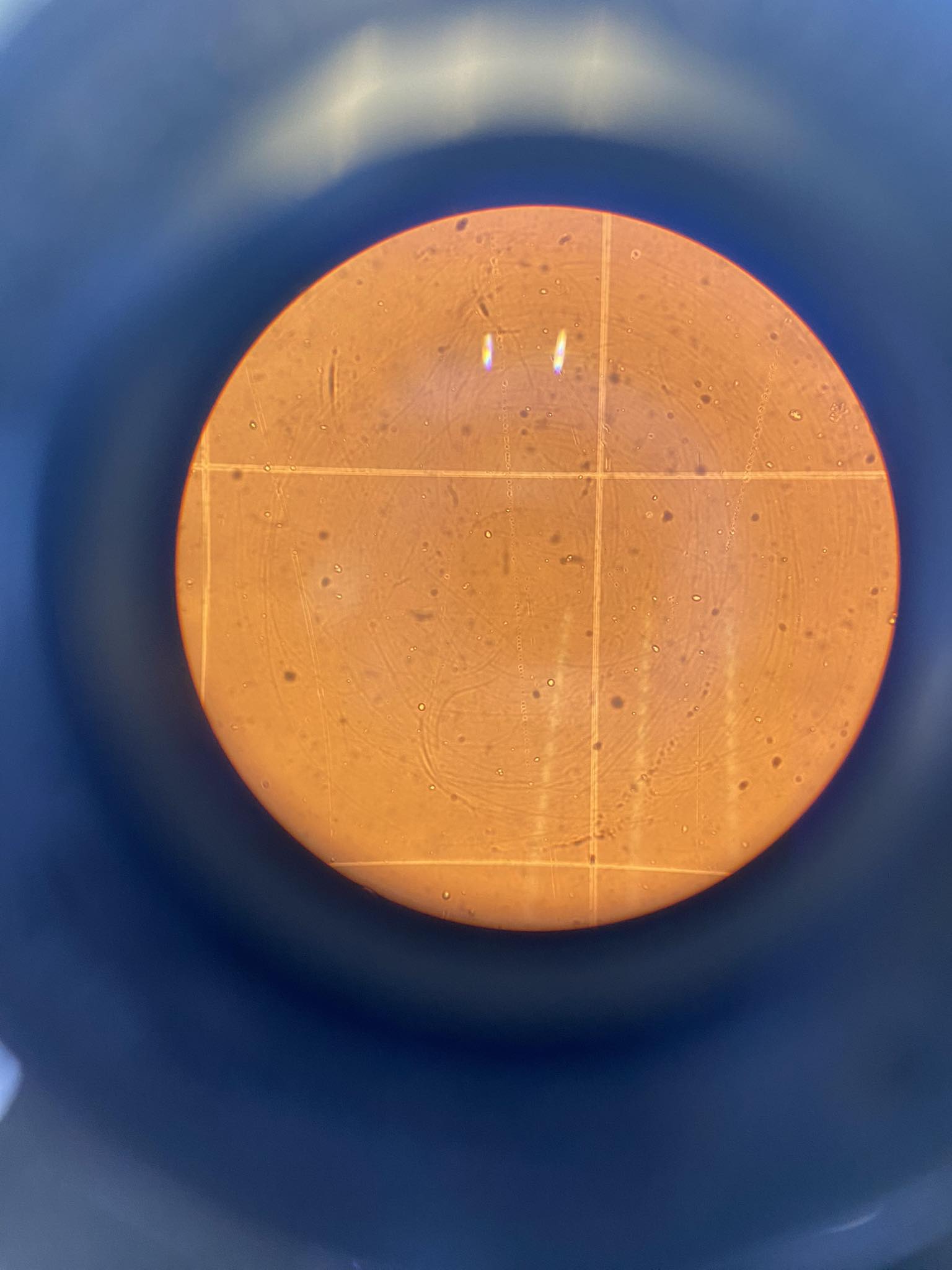

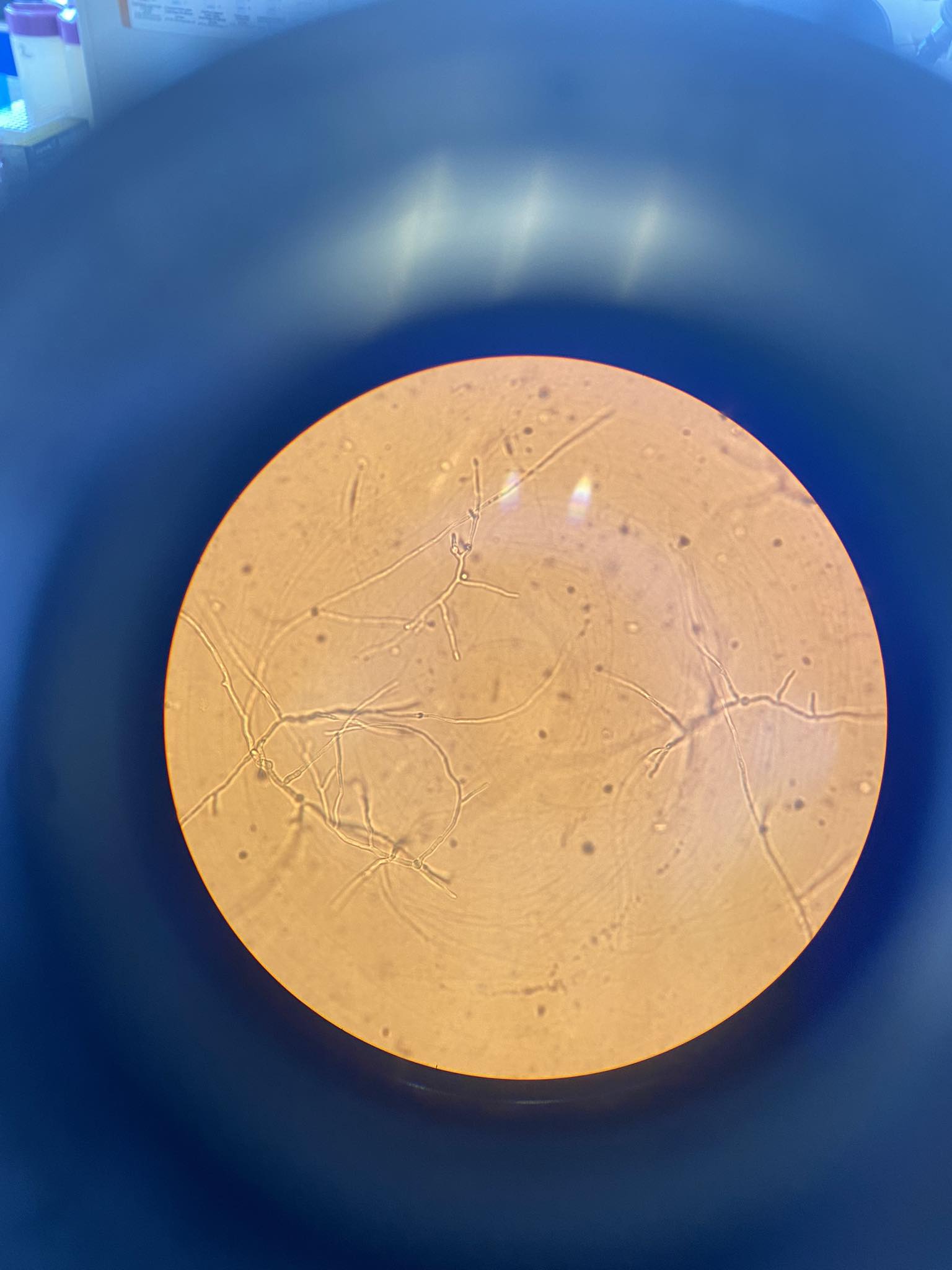


Control 1 mg×kg^-1^ 10 mg×kg^-1^ 100 mg×kg^-1^ 500 mg×kg^-1^

Control 1 mg×kg^-1^ 10 mg×kg^-1^ 100 mg×kg^-1^ 500 mg×kg^-1^

**Figure S1B:** Investigation of conidiation and hyphal development of *L. fungicola* 1722 when treated with fungicides prochloraz or metrafenone at 0, 1, 10, 100 and 500 mg×kg^-1^ for 24 hr. Evidence of conidiation and hyphal development was monitored using an Olympus microscope (40X).

**
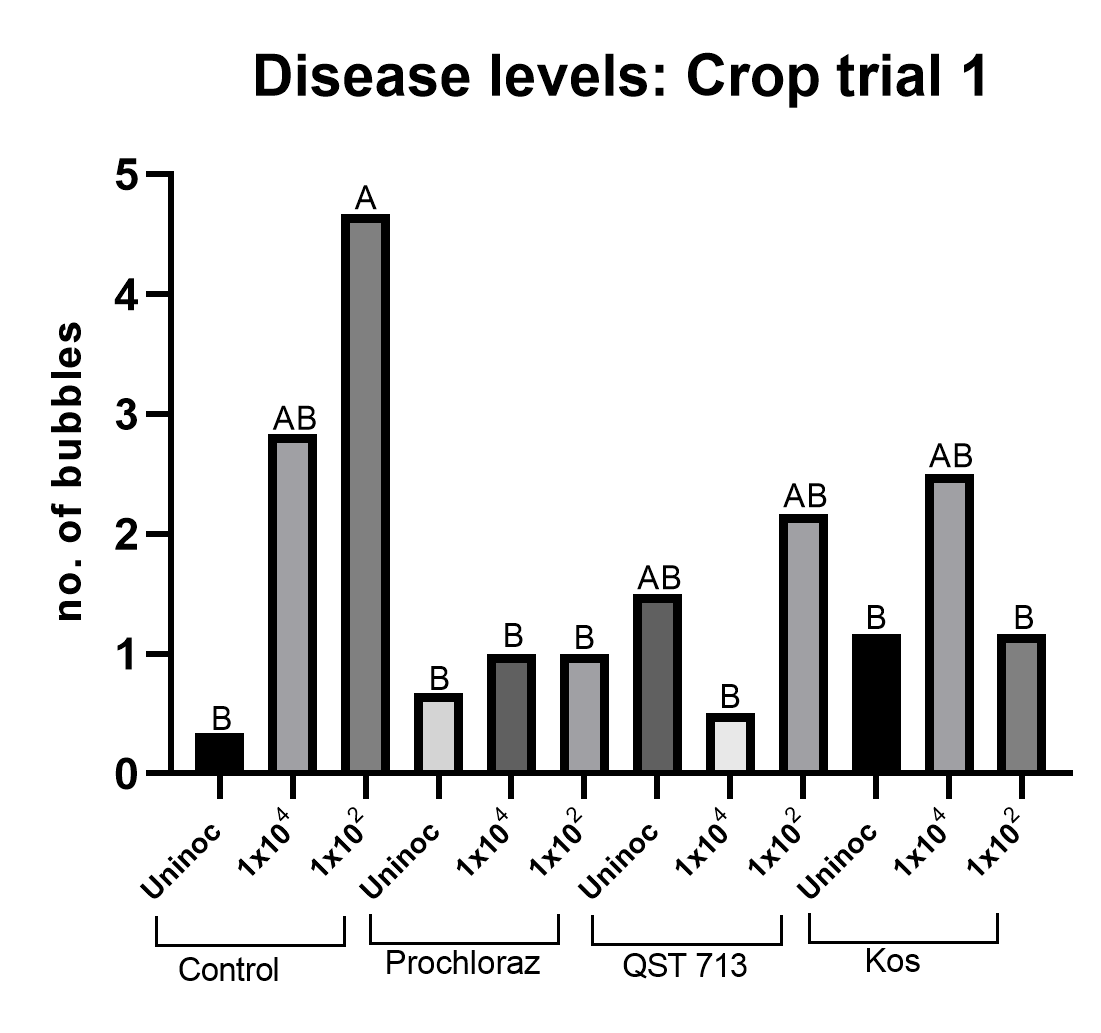
**

**Figure S2:** Average number of bubbles recorded at the end of crop trial 1 for plots treated fungicides Prochloraz or the BCAs QST 713 or Kos, followed by inoculation with 1×10^4^ conidia m^-2^ and 1× 10^2^ conidia m^-2^ *L. fungicola* 1722. Data analysed by ANOVA, n = 6. Means sharing the same letter are not significantly different at P <0.05 by Tukeys pairwise comparisons test.
