# Supplementary material for: The control of mushroom pathogen Lecanicillium fungicola with fungicides and Bacillus-based biocontrol treatments during crop trial studies
Source: BMC Microbiol. 2025 Nov 20;25:767. doi: 10.1186/s12866-025-04356-y (PMC12632097; doi:10.1186/s12866-025-04356-y)
Supplement: Supplementary file 2 — Supplementary Material 2. [file 12866_2025_4356_MOESM2_ESM.docx]

| **Treatment** | **Average weight of healthy mushrooms Flush 1** | **Average weight of healthy mushrooms Flush 2** | **Average weight of healthy mushrooms Flush 3** | **Average weight of healthy mushrooms, End of Trial** |
| --- | --- | --- | --- | --- |
|  |  |  |  |  |
| Control uninoculated | 4.30^A^ | 0.59 ^A^ | 1.30^A^ | 6.19^A^ |
| Control 1×10^6^ conidia m^-2^ | 4.51 ^A^ | 0.31 ^A^ | 0.44^C^ | 5.26^BCD^ |
| Control 1×10^4^ conidia m^-2^ | 4.3 ^A^ | 0.41 ^A^ | 1.22 ^A^ | 5.93^AB^ |
| Control 1×10^2^ conidia m^-2^ | 4.26 ^A^ | 0.63 ^A^ | 1.07^AB^ | 5.96^A^ |
| Prochloraz uninoculated | 4.55 ^A^ | 0.75 ^A^ | 1.17 ^A^ | 6.47^AB^ |
| Prochloraz 1×10^6^ conidia m^-2^ | 3.92 ^A^ | 0.55 ^A^ | 0.39^C^ | 4.86^D^ |
| Prochloraz 1×10^4^ conidia m^-2^ | 4.28 ^A^ | 0.49 ^A^ | 1.40 ^A^ | 6.17^A^ |
| Prochloraz 1×10^2^ conidia m^-2^ | 4.155 ^A^ | 0.63 ^A^ | 1.278 ^A^ | 6.06 ^AB^ |
| QST 713 uninoculated | 3.90 ^A^ | 0.84 ^A^ | 1.18 ^A^ | 5.92 ^AB^ |
| QST 713 1×10^6^ conidia m^-2^ | 4.187 ^A^ | 0.235 ^A^ | 0.46^BC^ | 4.88^CD^ |
| QST 713 1×10^4^ conidia m^-2^ | 4.09 ^A^ | 0.36 ^A^ | 1.31 ^A^ | 5.76 ^AB^ |
| QST 713 1×10^2^ conidia m^-2^ | 4.16 ^A^ | 0.39 ^A^ | 1.15 ^A^ | 5.70^ABC^ |
| Kos uninoculated | 3.96 ^A^ | 0.723 ^A^ | 1.252 ^A^ | 5.94 ^AB^ |
| Kos 1×10^6^ conidia m^-2^ | 4.12 ^A^ | 0.27 ^A^ | 0.39^C^ | 4.78^D^ |
| Kos 1×10^4^ conidia m^-2^ | 4.28 ^A^ | 0.49 ^A^ | 1.26 ^A^ | 6.03 ^AB^ |
| Kos 1×10^2^ conidia m^-2^ | 3.94 ^A^ | 0.40 ^A^ | 1.46 ^A^ | 5.80 ^AB^ |

**Supplementary Tables Table S1:** Average yield of healthy mushrooms (kg), crop trial 1

Data normally distributed and with equal variance analysed by ANOVA, n = 6. Means sharing the same letter are not significantly different at P <0.05 by Tukeys pairwise comparisons test.

**Table S2:** Disease levels, crop trial 1

| **1x10^6^ conidia m^-2^** | | | | |  |
| --- | --- | --- | --- | --- | --- |
|  |  |  |  |  |  |
| **Treatment** | **Average number of bubbles Flush 1** | **Average number of bubbles Flush 2** | **Average number of bubbles Flush 3** | **Total number of bubbles** |  |
|  |  |  |  |  |  |
| Control uninoculated | 0 | 0.2 | 0.2 | 0.333 |  |
| Control 1×10^6^ conidia m^-2^ | 3.5 | 29.8^A^ | 1.5 ^A^ | 34.83 **^A^** |  |
| Prochloraz uninoculated | 0 | 0.5 | 0.2 | 0.667 |  |
| Prochloraz 1×10^6^ conidia m^-2^ | 0.8 | 29.8^A^ | 1.2 ^A^ | 31.83 **^A^** |  |
| QST 713 uninoculated | 0 | 0.7 | 0.8 | 1.5 |  |
| QST 713 1×10^6^ conidia m^-2^ | 3.8 | 25 ^A^ | 0.7 ^A^ | 29.50 **^A^** |  |
| Kos uninoculated | 0 | 0.3 | 0.8 | 1.167 |  |
| Kos 1×10^6^ conidia m^-2^ | 4 | 31.7 ^A^ | 2.3 ^A^ | 38 **^A^** |  |
| **1x10^4^ conidia m^-2^ and 1x10^2^ conidia m^-2^** | | | | |  |
|  |  |  |  |  |  |
| **Treatment** | **Average number of bubbles Flush 1** | **Average number of bubbles Flush 2** | **Average number of bubbles Flush 3** | **Total number of bubbles** |  |
|  |  |  |  |  |  |
| Control uninoculated | 0 | 0.2 | 0.2 | 0.333 |  |
| Control 1×10^4^ conidia m^-2^ | 0 | 2.2 | 0.3 | 2.83 **^AB^** |  |
| Control 1×10^2^ conidia m^-2^ | 0 | 3.2 | 1.5 | 4.66**^A^** |  |
| Prochloraz uninoculated | 0 | 0.5 | 0.2 | 0.667 |  |
| Prochloraz 1×10^4^ conidia m^-2^ | 0.3 | 0.7 | 0 | 1**^B^** |  |
| Prochloraz 1×10^2^ conidia m^-2^ | 0 | 0.8 | 0.2 | 1**^B^** |  |
| QST 713 uninoculated | 0 | 0.7 | 0.8 | 1.5 |  |
| QST 713 1×10^4^ conidia m^-2^ | 0 | 0.5 | 0 | 0.5**^B^** |  |
| QST 713 1×10^2^ conidia m^-2^ | 0 | 1.2 | 1 | 2.166 **^AB^** |  |
| Kos uninoculated | 0 | 0.3 | 0.8 | 1.166 |  |
| Kos 1×10^4^ conidia m^-2^ | 0.5 | 1.3 | 1.3 | 2.5 **^AB^** |  |
| Kos 1×10^2^ conidia m^-2^ | 0 | 0.7 | 0.5 | 1.166**^B^** |  |

Data normally distributed and with equal variance analysed by ANOVA, n = 6. Uninoculated plots not included in ANOVA analysis. Means sharing the same letter are not significantly different at P <0.05 by Tukeys pairwise comparisons test.

**Table S3:** Average yield of healthy mushrooms (kg), crop trial 2

| **Treatment** | **Average weight of healthy mushrooms Flush 1** | **Average weight of healthy mushrooms Flush 2** | **Average weight of healthy mushrooms Flush 3** | **Average weight of healthy mushrooms, End of trial** |
| --- | --- | --- | --- | --- |
| Control uninoculated | 2.59 ^A^ | 2.18 ^A^ | 1.01 ^A^ | 5.78 ^A^ |
| Control 1×10^4^ conidia m^-2^ | 2.60 ^A^ | 1.97 ^A^ | 1.0 ^A^ | 5.57 ^A^ |
| Control 1×10^2^ conidia m^-2^ | 2.78 ^A^ | 2.08 ^A^ | 0.85 ^A^ | 5.71 ^A^ |
| Salted uninoculated | 2.68 ^A^ | 2.14 ^A^ | 1.15 ^A^ | 5.97 ^A^ |
| Salted 1×10^4^ conidia m^-2^ | 2.72 ^A^ | 2.02 ^A^ | 0.66 ^A^ | 5.40 ^A^ |
| Salted 1×10^2^ conidia m^-2^ | 2.75 ^A^ | 1.95 ^A^ | 1.03 ^A^ | 5.73 ^A^ |
| Metrafenone uninoculated | 2.44 ^A^ | 2.17 ^A^ | 0.95 ^A^ | 5.56 ^A^ |
| Metrafenone 1×10^4^ conidia m^-2^ | 2.61 ^A^ | 1.91 ^A^ | 0.94 ^A^ | 5.46 ^A^ |
| Metrafenone 1×10^2^ conidia m^-2^ | 2.76 ^A^ | 2.24 ^A^ | 1.18 ^A^ | 6.18 ^A^ |
| QST 713 uninoculated | 2.7 ^A^ | 2.06 ^A^ | 0.92 ^A^ | 5.68 ^A^ |
| QST 713 1×10^4^ conidia m^-2^ | 2.70 ^A^ | 2.08 ^A^ | 0.80 ^A^ | 5.58 ^A^ |
| QST 713 1×10^2^ conidia m^-2^ | 2.85 ^A^ | 1.85 ^A^ | 1.05 ^A^ | 5.75 ^A^ |
| Kos uninoculated | 2.72 ^A^ | 2.03 ^A^ | 1.06 ^A^ | 5.81 ^A^ |
| Kos 1×10^4^ conidia m^-2^ | 2.46 ^A^ | 2.22 ^A^ | 0.86 ^A^ | 5.54 ^A^ |
| Kos 1×10^2^ conidia m^-2^ | 2.5 ^A^ | 2.26 ^A^ | 0.96 ^A^ | 5.72 ^A^ |

Data normally distributed and with equal variance analysed by ANOVA, n = 6. Means sharing the same letter are not significantly different at P <0.05 by Tukeys pairwise comparisons test.

**Table S4:** Disease levels, crop trial 2

| **1x10^4^ conidia m^-2^** | | | | |  |
| --- | --- | --- | --- | --- | --- |
| **Treatment** | **Average number of bubbles Flush 2** | **Average number of bubbles Flush 3** | **Total number of bubbles** | **Efficacy end of trial** |  |
|  |  |  |  |  |  |
| Control uninoculated | 0 | 0.2 | 0.2 |  |  |
| Control 1×10^4^ conidia m^-2^ | 3.2 ^A^ | 13.6 ^A^ | 16.8 ^A^ |  |  |
| Salted uninoculated | 0 | 0.2 | 0.2 |  |  |
| Salted 1×10^4^ conidia m^-2^ | 3.4 ^A^ | 1.2 ^B^ | 4.6 ^B^ | 73% |  |
| Metrafenone uninoculated | 0 | 0 | 0 |  |  |
| Metrafenone 1×10^4^ conidia m^-2^ | 0.2^B^ | 0.4 ^B^ | 0.6 ^B^ | 96% |  |
| QST 713 uninoculated | 0 | 0 | 0 |  |  |
| QST 713 1×10^4^ conidia m^-2^ | 0 ^B^ | 2.4 ^B^ | 2.4 ^B^ | 86% |  |
| Kos uninoculated | 0 | 0 | 0 |  |  |
| Kos 1×10^4^ conidia m^-2^ | 1.4 ^AB^ | 3 ^B^ | 4.4 ^B^ | 74% |  |
| **1x10^2^ conidia m^-2^** | | | | |  |
|  | **Average number of bubbles Flush 2** | **Average number of bubbles Flush 3** | **Total number of bubbles** |  |  |
|  |  |  |  |  |  |
| Control uninoculated | 0 | 0.2 | 0.2 |  |  |
| Control 1×10^2^ conidia m^-2^ | 0 | 1.5 | 1.5 |  |  |
| Salted uninoculated | 0 | 0.2 | 0.2 |  |  |
| Salted 1×10^2^ conidia m^-2^ | 0 | 0 | 0 |  |  |
| Metrafenone uninoculated | 0 | 0 | 0 |  |  |
| Metrafenone 1×10^2^ conidia m^-2^ | 0 | 0 | 0 |  |  |
| QST 713 uninoculated | 0 | 0 | 0 |  |  |
| QST 713 1×10^2^ conidia m^-2^ | 0 | 0 | 0 |  |  |
| Kos uninoculated | 0 | 0 | 0 |  |  |
| Kos 1×10^2^ conidia m^-2^ | 0 | 0 | 0 |  |  |

Data normally distributed and with equal variance analysed by ANOVA, n = 5. Uninoculated plots not included in ANOVA analysis. Means sharing the same letter are not significantly different at P <0.05 by Tukeys pairwise comparisons test

**Table S5:** Average yield of healthy mushrooms (kg), crop trial 3

| **Treatment** | **Average weight of healthy mushrooms Flush 1** | **Average weight of healthy mushrooms Flush 2** | **Average weight of healthy mushrooms, End of trial** |
| --- | --- | --- | --- |
| Control uninoculated | 1.99 ^A^ | 2.06 ^A^ | 4.05^A^ |
| Control 1×10^2^ conidia m^-2^ | 1.78 ^A^ | 2.39 ^A^ | 4.17^A^ |
| Control 1×10^4^ conidia m^-2^ | 2.17 ^A^ | 1.95 ^A^ | 4.12^A^ |
| Control 1×10^6^ conidia m^-2^ | 2.02 ^A^ | 0.85 ^B^ | 2.87^B^ |
| Salted uninoculated | 1.99 ^A^ | 2.11 ^A^ | 4.10^A^ |
| Salted 1×10^4^ conidia m^-2^ | 2.05 ^A^ | 2.08 ^A^ | 4.13^A^ |
| Metrafenone uninoculated | 2.05 ^A^ | 2.30 ^A^ | 4.35^A^ |
| Metrafenone 1×10^4^ conidia m^-2^ | 2.08 ^A^ | 2.22 ^A^ | 4.30^A^ |
| QST 713 uninoculated | 2.35 ^A^ | 2.16 ^A^ | 4.50^A^ |
| QST 713 1×10^4^ conidia m^-2^ | 2.02 ^A^ | 2.19 ^A^ | 4.21^A^ |
| Kos uninoculated | 1.77 ^A^ | 2.53 ^A^ | 4.29^A^ |
| Kos 1×10^4^ conidia m^-2^ | 1.74 ^A^ | 2.56 ^A^ | 4.30^A^ |

Data normally distributed and with equal variance analysed by ANOVA, n = 6. Means sharing the same letter are not significantly different at P <0.05 by Tukeys pairwise comparisons test.

**Table S6:** Disease levels, crop trial 3

| **Treatment** | **Average number of bubbles Flush 1** | **Average number of bubbles Flush 2** | **Total number of bubbles** |
| --- | --- | --- | --- |
| Control uninoculated | 0 | 0.167 | 0.167 |
| Control 1×10^4^ conidia m^-2^ | 0.167 | 11.17 ^A^ | 11.33 ^A^ |
| Salted uninoculated | 0 | 0 | 0 |
| Salted 1×10^4^ conidia m^-2^ | 0.167 | 3.5 ^B^ | 3.67 ^B^ |
| Metrafenone uninoculated | 0 | 0 | 0 |
| Metrafenone 1×10^4^ conidia m^-2^ | 0 | 2.83 ^B^ | 2.83 ^B^ |
| QST 713 uninoculated | 0 | 0.167 | 0.167 |
| QST 713 1×10^4^ conidia m^-2^ | 0 | 3.0 ^B^ | 3.0 ^B^ |
| Kos uninoculated | 0 | 0 | 0 |
| Kos 1×10^4^ conidia m^-2^ | 0.167 | 1.67 ^B^ | 1.83 ^B^ |
|  | | | |
| Control uninoculated | 0 | 0.167 | 0.167 |
| Control 1×10^6^ conidia m^-2^ | 3.83 ^A^ | 84.33 ^A^ | 88.2 ^A^ |
| Control 1×10^4^ conidia m^-2^ | 0.167 ^B^ | 11.17 ^B^ | 11.33 ^B^ |
| Control 1×10^2^ conidia m^-2^ | 0 ^B^ | 0 ^B^ | 0 ^B^ |

Data normally distributed and with equal variance analysed by ANOVA, n = 6. Uninoculated plots not included in ANOVA analysis. Means sharing the same letter are not significantly different at P <0.05 by Tukeys pairwise comparisons test.
